# Effects of Holistically Conceptualised School-Based Interventions on Children’s Physical Literacy, Physical Activity, and Other Outcomes: A Systematic Review

**DOI:** 10.1186/s40798-024-00766-w

**Published:** 2024-09-27

**Authors:** Alethea Jerebine, Lauren Arundell, Kimberley Watson-Mackie, Richard Keegan, Petra Jurić, Dean Dudley, Nicola D. Ridgers, Jo Salmon, Lisa M. Barnett

**Affiliations:** 1 School of Health and Social Development, Deakin University, Melbourne, VIC 3125 Australia; 2 Centre for Sport, Exercise and Life Sciences, Coventry University, Coventry, UK; 3 School of Exercise and Nutrition Sciences, Deakin University, Melbourne, VIC Australia; 4 Institute for Physical Activity and Nutrition, Deakin University, Melbourne, VIC Australia; 5 Research Institute for Sport and Exercise (UCRISE), Faculty of Health, University of Canberra, Canberra, ACT Australia; 6 Faculty of Kinesiology, University of Zagreb, Horvaćanski zavoj 15, Zagreb, 10 000 Croatia; 7 Macquarie School of Education, Macquarie University, Sydney, NSW Australia; 8 Alliance for Research in Exercise, Nutrition and Activity (ARENA), Allied Health and Human Performance, University of South Australia, Adelaide, SA Australia

**Keywords:** Child, Adolescent, Physical education, Motor skills

## Abstract

**Background:**

Schools are a key setting for promoting children’s physical literacy development. This review aimed to identify school-based interventions that adopted a holistic conceptualisation of physical literacy and examine the effects on children’s physical literacy and any other outcomes, including physical activity (PA).

**Methods:**

Searches were conducted in seven databases (APA PsycINFO, EMBASE, ERIC, CINAHL, Global Health, MEDLINE Complete, SPORTDiscus with Full Text), and Google and Google Scholar, to identify articles published since 1/1/2017. Studies were included if they (i) adopted a holistic conception of physical literacy as represented by the Australian Physical Literacy Framework (APLF), (ii) were grounded in movement, (iii) assessed three or more domains of learning (either quantitatively or qualitatively), and (iv) included children aged 5–14 years. Quantitative research designs needed to provide pre-and post-intervention measures, whereas qualitative designs (e.g. post-intervention interviews) did not. Study selection, data extraction and quality assessment were conducted independently by teams of two authors. For intervention effects, quantitative and qualitative data were synthesised separately. For quantitative data, level of evidence for intervention effects was assessed by physical literacy domain and/or elements/items by examining the proportion of tests with a significant change in the expected direction. Qualitative data were synthesised using the framework synthesis method and mapped to a framework that included APLF domains/elements, PA, and additional outcomes.

**Results:**

Twelve interventions with 1,427 participants from seven countries were identified: six physical education-based, three afterschool, one structured recess, and two multicomponent. All studies assessed the physical domain quantitatively, with strong positive evidence of intervention effects for the controlled designs (10 of 15 tests). For the affective and cognitive domains, evidence was mixed, and there was no evidence for interventions improving the social components of children’s physical literacy (although this was understudied). Most studies assessed PA and one measured cognitive performance; however, there was no evidence for positive intervention effects (i.e. ≥35% of tests reporting an improvement) for either outcome. Five studies assessed intervention effects qualitatively, with positive results reported for all physical literacy domains, PA, and cognitive performance.

**Conclusions:**

Holistic interventions in schools can improve the physical domain of children’s physical literacy. For wider benefits, future interventions should aim to develop all facets of physical literacy, especially domains of learning less frequently targeted and examined.

**Trial Registration:**

PROSPERO CRD42022351317.

**Supplementary Information:**

The online version contains supplementary material available at 10.1186/s40798-024-00766-w.

## Key Points

This review examined the effects of holistically conceptualised school-based interventions on (i) children’s physical literacy across physical, affective, cognitive, and social domains of learning, and (ii) any other outcomes.

There was strong positive evidence of improvements in the physical domain. There was mixed evidence for holistic physical literacy interventions improving total physical literacy or the affective and cognitive domains of physical literacy.

There was no evidence for holistic physical literacy interventions improving the social components of children’s physical literacy or other outcomes such as physical activity or cognitive performance, although qualitative findings reported benefits across all domains and for physical activity.

**Supplementary Information:**

The online version contains supplementary material available at 10.1186/s40798-024-00766-w.

## Background

Physical literacy is gaining traction worldwide as a concept that seeks to provide a holistic understanding of the skills and capacities individuals require to lead active lives across the lifespan [1–4]. Physical literacy can be developed at any age, but childhood is recognised as a crucial period for the accumulation of movement experiences that contribute to physical literacy [5]. Moreover, emerging evidence suggests physical literacy plays a formative role in shaping physical activity (PA) trajectories from a young age [6]. Although considerable differences exist in how the physical literacy construct is defined and applied [7–9], nations across North and South America, Europe, Asia and the Pacific have adopted the term in policy directives for education and health promotion [4, 10–12].

Of the various definitions internationally, in this review we have used the Australian definition: “*Physical literacy is lifelong holistic learning acquired and applied in movement and physical activity contexts*” [4, 13]. A unique aspect of the Australian definition is its holistic interconnected conceptualisation in the Australian Physical Literacy Framework (APLF), which encompasses four domains [physical, psychological (hereafter termed ‘affective’), social and cognitive] and 30 elements [4]. We have adopted the term ‘affective’ for the domain originally termed ‘psychological’ in the APLF, to both align with recent developments in physical literacy literature [14–16] and reflect that within the psychology discipline there are several subdomains which include affect/emotion (affective), as well as social psychology, and cognition, among others. We note here the potential for referring to the physical domain of physical literacy as ‘psychomotor’ (the integration of cognitive and physical learning) to be consistent with learning and education literature [17–19]. However, we have kept the term ‘physical’ as this is commonly used in the literature on physical literacy [14, 16, 20]. The four domains of the APLF underscore the holistic nature of physical literacy, emphasising the interconnectedness of physical, mental, and social wellbeing.

There is a rapidly evolving evidence base for interventions seeking to develop physical literacy and/or assess physical literacy outcomes, across the lifespan, and particularly in children. Schools provide an ideal setting for interventions seeking to engage in and promote children’s physical literacy development, as most children spend a substantial portion of their day at school, with multiple opportunities to engage in learning through movement over the school day, including physical education (PE), recess/breaktimes, active/outdoor classrooms, and free time before and after school [21–24]. Although the literature on the effectiveness of physical literacy interventions has been subject to several recent reviews [15, 25–28], to our knowledge, none have sought to synthesise school-based interventions that adopt a holistic conceptualisation of physical literacy. To our knowledge previous reviews have not applied a holistic conceptualisation of physical literacy in their eligibility criteria [15, 25–28]. Consequently, these reviews have synthesised a broad range of interventions with respect to how physical literacy is defined, embedded in intervention content, and assessed, including studies with a narrow interpretation of physical literacy as just fundamental movement skills. A 2022 review identified that although most (77%) interventions (*n* = 46) adopted a holistic understanding of physical literacy (according to their definition), only 39% had research goals or intervention content for three or more physical literacy domains [20]. As such there may be a lack of understanding in the field regarding the effects of interventions that seek to develop physical literacy holistically across the physical, affective, cognitive, and social domains. Additionally, previous evidence syntheses have combined physical literacy interventions across population age groups and settings, which may limit the practical applicability of their findings.

Given the significance of the school-setting in providing children with multiple opportunities to engage in and promote physical literacy development, and the expanding research in this field, this review aimed to examine: (i) the effects of holistically conceptualised school-based interventions on the physical literacy of children aged 5–14 years across three or more domains of learning, and (ii) whether any other outcomes such as PA were improved. As measurement of physical literacy is an emerging field, with many approaches implemented globally, including objective, self-report and proxy-report, qualitative study designs were included (in addition to quantitative designs) where participants described physical and movement skill experiences and outcomes after participating in interventions that aimed to develop children’s physical literacy.

## Methods

### Review Design

We used the convergent segregated approach to mixed-methods systematic reviews, which involved an independent synthesis of quantitative data and qualitative data followed by the integration and configuration of evidence using the framework synthesis method [29]. The APLF was selected as a holistic theoretical framework against which to map and configure the findings from included studies [30, 31]. Due to the degree of heterogeneity in study designs and physical literacy assessment methods employed in studies identified through the systematic search, meta-analysis of quantitative evidence was not feasible. The review was conducted as part of a wider project commissioned by the Australian Sport Commission. We prospectively registered the review (CRD:42022351317) with the International Prospective Register of Systematic Reviews (PROSPERO) and followed the 2020 guidelines of the Preferred Reporting Items for Systematic Reviews and Meta-Analyses (PRISMA) [32]. Please see Table S1 in the Electronic Supplementary Material (ESM) for the PRISMA checklist.

### Searches

The search for relevant literature was conducted in two steps. First, we conducted a systematic search across seven scientific databases (APA PsycINFO, EMBASE, ERIC, CINAHL Complete, Global Health, MEDLINE Complete, SPORTDiscus with Full Text) to identify studies or reviews (narrative or systematic) of school-based physical literacy interventions. The search strategy, which combined terms for ‘child, ‘school’, ‘physical literacy’, and ‘intervention’, was developed and adapted for each database by a university health librarian with expertise in advanced database searching. The first search was conducted on 25 July 2022 and restricted to peer-reviewed English language articles published since 1 January 2017. This timeframe was selected to synthesise the most recent evidence on holistic physical literacy interventions. In a previous review of 48 physical literacy interventions in multiple settings and populations, only five were conducted before 2017 and none of these were holistically conceptualised, school-based programs [15]. An updated database search was conducted on 14 November 2023. Please see Table S2 in the ESM for the final search plan. Second, we conducted searches through *Google* and *Google Scholar* under the guidance of the university health librarian. For the search of *Google*, a search string of keywords combining the terms ‘physical literacy’, ‘school’, and ‘program’ was developed to identify any studies or interventions not returned in the database search. Next, the terms ‘physical literacy’ and ‘reviews’ were combined with date and keyword parameters using the advanced search function in *Google Scholar* to identify suitable reviews of physical literacy interventions. Both *Google* searches were also limited to records published since 1 January 2017, performed on 12 August 2022, and updated on 21 November 2023.

### Study Screening and Selection

Screening aimed to identify: (a) any reviews of physical literacy interventions published since 1 January 2017; or (b) any articles reporting the outcomes of school-based interventions that sought to develop the physical literacy of children aged 5 to 14 years old. All records identified in the database searches were imported into Endnote™ where duplicates were removed before exporting to Covidence™ software. In Covidence™, remaining records were independently screened against pre-defined eligibility criteria (see Table 1) by teams of two authors, with discrepancies resolved through discussion with the wider author team. Records identified in the *Google* and *Google Scholar* searches were screened manually by one author. Screened records from the *Google* and *Google Scholar* searches were imported into an MS Excel file and discussed with the wider author team. Reference lists of all included articles were hand-searched for further relevant studies.

Following title and abstract screening, the selection of studies for inclusion in the review was conducted in two stages. As this is a rapidly evolving area of research and there is considerable variation in definitions and application of physical literacy internationally, in the 1st stage of full-text screening we included all definitions and measures of physical literacy. However, since the aim of this review was to identify interventions that adopted *a holistic*,* interconnected conception of physical literacy*, at the 2nd stage of full-text screening, only interventions that were grounded in movement and assessed three or more domains of learning (as represented in the APLF or other physical literacy definitions [33]) were included in the evidence synthesis. For quantitative research designs, studies were included if physical literacy outcomes were measured pre-and post-intervention, whereas for qualitative research designs, cross-sectional methods, such as post-intervention interviews, were also eligible.

**Table 1:** Eligibility criteria for study screening and selection

Inclusion criteria	− Language: Published in English − Article Type: Original research and reviews (narrative or systematic)
	− Sample: Children (typically developing or not) with a reported mean age or age range between 5–14 years who are attending school
	− Setting: School, e.g., primary, elementary, middle, secondary. Includes before/after-school programs conducted in schools
	1st stage full-text screening − Intervention: school-based interventions seeking to develop children’s physical literacy (all definitions and applications included)
	2nd stage full-text screening − Intervention: grounded in movement − Outcome: Physical literacy outcomes, assessed either quantitatively or qualitatively, for at least 3 domains of learning consistent with the APLF − Quantitative methods: study designs included randomised controlled trials, pre-post with controls, pre-post without controls, and pilot or preliminary, if they met the pre-post requirement. − Qualitative methods: all designs considered, either pre-post-assessment or post-assessment only
Exclusion criteria	− Articles that do not report empirical data collection e.g., protocols, conceptual articles, opinion pieces
	− Settings other than school such as early childhood, outdoor education camps, community-based sport, or before/after-school programs conducted outside of schools
	− Study sample does not include children aged 5 to 14 years old e.g., only teachers, senior high school students
	− Study does not report physical literacy outcomes of a school-based intervention e.g., cross-sectional observation designs measuring associations between physical literacy and other variables
	− Studies that employ and/or measure a narrowly conceptualised definition of physical literacy e.g., less than 3 APLF domains

### Data Extraction

Data extraction was performed in five stages: (1) descriptive data for included studies; (2) intervention characteristics and alignment with a holistic physical literacy definition; (3) risk of bias assessment; (4) methods and tools for measuring physical literacy and other outcomes; and (5) intervention outcomes. Each stage is described below. All data extraction was conducted by a minimum of two authors, with any discrepancies discussed with the wider author team to ensure consistency in interpretation and extraction.

#### Descriptive Data

Using customised data extraction templates, we extracted the following descriptive data for included studies: publication date, country, study setting and population, intervention design and school context, data collection methods, physical literacy and other outcome assessment tools, and summary outcomes.

#### Intervention Characteristics and Alignment with a Holistic Physical Literacy Definition

To further delineate how physical literacy was conceptualised and construed, the aims and content of each intervention were examined for alignment with the physical literacy definition and/or compatible philosophical assumptions stated in the study. Using a customised template, we extracted the following data: physical literacy definition, intervention content, physical literacy domains of learning targeted, alignment between physical literacy definition and intervention content, and intervention delivery characteristics. Studies were assessed as strongly, moderately, or weakly aligned. A strongly aligned intervention demonstrated a consistent flow from the physical literacy definition, intervention aims and content through to the physical literacy outcomes assessed, a moderately aligned intervention showed some alignment but not all domains were explained, and weakly aligned interventions demonstrated little or no consistency, for example, description of intervention aims and content did not correspond to the physical literacy domains assessed.

#### Risk of Bias

Two reviewers (authors NR and PJ) independently and blindly assessed the risk of bias of all studies that met our inclusion criteria, with any disagreements resolved through discussion. For quantitative methodology, assessment contained the following domains, adapted from van Sluijs et al. [34]: (1) baseline characteristics comparable; (2) randomisation clearly described and carried out; (3) validated measures of physical literacy used; (4) dropout described; (5) dropout ≤ 20% for < 6-month (m) follow-up and ≤ 30% for ≥ 6-m follow-up; (6) outcome assessors blinded; (7) sample size/power calculation reported; (8) study adequately powered; (9) intention to treat analysis used; (10) potential confounders accounted; and (11) participants followed for ≥ 6-m. In each of the domains, studies were assigned as: (1) positive – confirms that the domain criterion was met; (2) negative – refutes that the domain criterion was met; (3) not or insufficiently described – information encompassing the domain was not present; or (4) not applicable – information encompassing the domain was not in accordance with the study.

For qualitative methodology, the following domains were assessed using the Critical Appraisal Skills Programme [35] (CASP) Checklist: (1) clear statement of aims; (2) qualitative methodology appropriate; (3) design appropriate to the aims; (4) clear and consistent theoretical underpinnings; (5) appropriate recruitment strategy; (6) data collected in a way that addressed the research; (7) relationship between researcher and participants; (8) ethical issues considered; (9) data analysis sufficiently rigorous; and (10) clear statement of findings. In each of the domains, studies were assigned as: (1) yes – confirms that the domain criterion was met; (2) no – refutes that the domain criterion was met; (3) somewhat – the domain criterion was partly met; or (4) cannot tell – information encompassing the domain was not clear to reviewers.

#### Assessment Methods and Tools for Measuring Physical Literacy and Other Outcomes

Using a customised template, the assessment instruments for included studies (quantitative designs) were classified against the APLF by domain and data collection method, including: (1) physical domain (self-report and observation measures); (2) affective domain (self-report); (3) cognitive domain (self-report); and (4) social domain (self-report). All studies assessed PA either as a behavioural domain of physical literacy or additional outcome (self-report and device-based measures). One study assessed an outcome other than physical literacy (as they defined it), specifically cognitive performance (objective measures). Assessment instruments for these items were also extracted. Data collection methodology employed in studies that explored intervention outcomes qualitatively were extracted in a second template and included study design, qualitative data collection method(s), participants, qualitative research aims/questions, data analysis methods, and approach to trustworthiness/rigour.

#### Physical Literacy and Other Intervention Outcomes

For each study, we extracted reported results for physical literacy and other intervention outcomes and mapped them in a customised template to either the APLF, a behavioural domain of physical literacy that included PA, or a wider outcome of interest. For controlled interventions that reported both within and between group x time results, we extracted only between group x time. For uncontrolled designs, within group x time results were extracted. For interventions that reported multiple timepoints (e.g., post-intervention and follow up results), results from all timepoints were extracted. Likewise, for interventions with two or more intervention arms, the results for each arm were extracted. For qualitative findings, both participant accounts (i.e., quotes) and author interpretation were labelled descriptively, then mapped to the APLF or other outcomes. Participants’ accounts included both child self-reported outcomes as well as teacher- or parent-observed outcomes.

### Data Synthesis

To examine the effect of interventions on different domains of learning, quantitative and qualitative data were synthesised separately. For quantitative data, intervention effects were synthesised by study design. Depending on the physical literacy instrument used, some studies provided an overall physical literacy score, while others reported outcomes by domain and/or element. Intervention effect was reported as either an improvement, as indicated by a significant result or effect size in the expected direction, or no/non-significant improvement, as indicated by a non-significant result, no effect, or result not in the expected direction (with too few results to group these results separately). Using a method developed by Lubans et al. [36] and Page et al. [37], the level of evidence for intervention effects was assessed for each physical literacy domain, PA or other outcome by examining the proportion of tests with a significant improvement in the expected direction. Physical literacy domains and outcomes were rated as follows: ‘0’ where less than 35% of tests found a significant improvement; ‘?’ where 35–59% of tests found an improvement; ‘+’ where 60–100% of tests found an improvement; and ‘++’ where 60–100% of tests found an improvement in four or more studies. Where a study used multiple tests and assessments for different domains and/or elements, these were reported individually (not by study).

Qualitative data were synthesised using framework synthesis, which is a structured but flexible method for integrating and interpreting diverse evidence from multiple studies [38]. Following the five stages of framework synthesis (familiarisation, framework selection, indexing, charting, mapping and interpretation), analysis began with *familiarisation*, whereby included studies were read several times by two reviewers (authors AJ and RK). Secondly, a *framework was selected* (the APLF domains and elements) to guide the analysis [38, 39]. As many studies assessed PA, a coding category for PA outcomes was added to the framework. The operational definitions for each coding category in the framework were based on the APLF and a broad definition of PA was adopted [40] to include any active behaviour, including play and sports. In the third stage, qualitative outcome data reported in included studies as ‘results’ or ‘findings’ were extracted, *indexed*, and labelled by two authors working independently. New coding categories were created where intervention outcomes did not fit the initial coding framework [38, 39]. Next, codes and labels were *charted*, discussed between authors, and refined into themes. These themes were then *mapped* to an updated framework, that included APLF domains and elements, PA, and additional outcomes, which was used to produce the manuscript [39]. The trustworthiness of our analysis was enhanced by the systematic application of the framework synthesis method, and researcher triangulation, whereby two authors undertook an iterative process of coding, labelling, discussion, and refinement of themes, which were then presented to the wider research team for discussion [38]. In the final stage of synthesis, the quantitative and qualitative syntheses were combined to provide a holistic understanding of the effectiveness of school-based physical literacy interventions.

## Results

### Study Selection

**Fig. 1:**
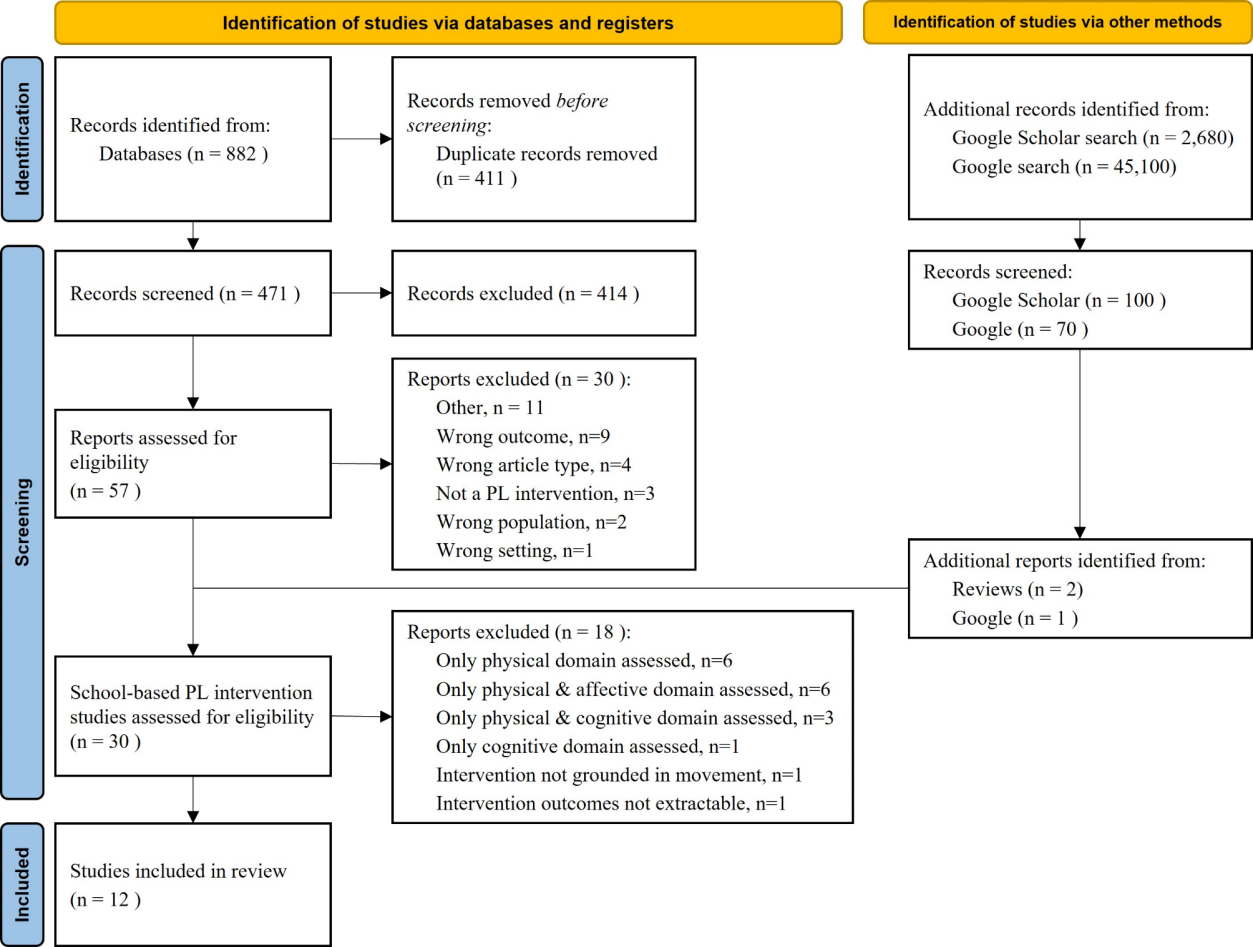
PRISMA flowchart of included studies. ‘Other’ includes no full text, duplicates, not published in English. *Abbreviations* PL = physical literacy

The systematic database search yielded 623 studies in July 2022, and a further 259 in the updated search in November 2023. After duplicate records were removed, there were 471 records for screening. Of these, 414 were excluded at the title abstract stage, leaving 57 for full-text screening. Ten previously published reviews were identified through the database and *Google Scholar* searches, which were also screened for potential inclusions (see Table S3 of the ESM) [15, 20, 25–28, 41–44]. At the first full-text screening stage, 27 school-based physical literacy intervention studies were identified through the database searches, two additional studies were identified from the previously published reviews [45, 46], and one further study through the *Google* search [47]. During the second full-text screening stage, 18 studies were excluded by consensus, leaving a final total of 12 studies for inclusion. The studies excluded at the second screening stage are detailed in Table S4 of the ESM. The complete search record, including reasons for exclusion of studies at the first and second full-text screening stage, is detailed in the PRISMA diagram in Fig. 1.

### Description of the Final Sample of School-Based Physical Literacy Intervention Studies

Half of the included studies were conducted in Canada (*n* = 6) [46–51], with the remaining six conducted in six different countries: Australia [52], Hong Kong [53], Italy [45], Portugal [54], Spain [55], and the USA [56]. Two thirds (*n* = 8) were published since 2020. Most interventions were conducted with primary/elementary school students except for two studies, one of which was conducted with a mix of primary and early secondary students [54], and the other with early secondary school students [56]. All were conducted with typically developing children; no school-based physical literacy interventions were identified that included children living with disability or a medical condition.

With respect to the type of school-based intervention, most (*n* = 6) were implemented during physical education (PE) [45, 47, 49, 50, 54, 56], with a further three being afterschool programs [46, 48, 51], one structured recess program [55], and two multi-component interventions involving a combination of PE, active lessons, and/or active games during recess [52, 53]. A variety of research designs were employed, including randomised controlled trials (*n* = 4) [45, 46, 52, 53], quasi-experimental controlled trials (*n* = 3) [47, 49, 55], within-subjects design (pre-post) (*n* = 3) [48, 50, 56], a mixed methods evaluation study [51], and a participatory case study with retrospective evaluation [54].

Study sample sizes also varied; three interventions had a sample of 200 participants or more [49, 50, 52], two had between 100 and 200 participants [45, 47], three had between 50 and 100 participants [46, 53, 55], and four had less than 50 participants [48, 51, 54, 56]. Most studies assessed physical literacy quantitatively, with four employing a mix of quantitative and qualitative methods [45, 51, 52, 56]. However, one of these, the cross-sectional study design of Caldwell, Miller [51] meant the quantitative data did not meet the eligibility criteria for this review. Additionally, one study assessed physical literacy outcomes qualitatively only, using a multi-method approach [54]. A complete description of included studies is provided in Table S5 of the ESM.

### Intervention Characteristics and Alignment with a Holistic Physical Literacy Definition

Although all studies adopted a holistic definition or interpretation of physical literacy, they varied in how the intervention content aligned with a multidimensional understanding of the construct (see Table 2). Nine of the 12 interventions demonstrated strong alignment between their adopted definition and the intervention or program components and/or pedagogical approach [45–49, 52–54, 56], two showed moderate alignment [50, 55], and one showed weak alignment [51]. Of the strongly aligned interventions, three were established PE programs grounded in a holistic pedagogical approach consistent with physical literacy development, such as circus arts instruction [49], the *Teaching Games for Understanding* (TGfU) based PlaySport program [48], and the *Sport Education* model [54]. In contrast, other strongly aligned interventions were newly developed programs that incorporated various pedagogical strategies to support children’s physical literacy development across multiple domains of learning [45–47, 52, 53, 56]. For example, the PE intervention, PLitPE, was a movement skills intervention based on the learning principles of repetition of skills and knowledge of results, that integrated augmented feedback and positive challenges consistent with Self-Determination Theory [47]. The authors of that study hypothesised that through linking physical competence to the knowledge of results, participants would simultaneously develop competence (physical domain), knowledge of movement terminology (cognitive domain), as well as fostering confidence and motivation (affective domain) through fun and social engagement. In contrast, the weakly aligned intervention evaluated physical literacy outcomes of an established PA program without reporting how the program was designed to support children’s holistic physical literacy development across the domains assessed [51].

**Table 2:** Intervention characteristics and alignment with a holistic physical literacy definition

Study, location	School setting	PL definition adopted	Intervention content (year IV conducted, if reported)	PL domains of learning targeted^a^				Intervention alignment	Intervention delivery characteristics				
				Phys	Psych	Cogn	Soc		Length	Frequency	Duration (per session)	Provider	Provider Training
Bremer et al. (2020) [46] Canada	Afterschool program	None provided Authors note “*physical literacy is a multidimensional concept that includes domains of movement competence*,* positive affect*,* confidence*,* and motivation necessary for regular engagement of physical activity*” Edwards et al. (2017) [1]	Each PL domain targeted through multiple teaching and intervention strategies: FMS stations and active games grounded in a mastery approach; mentoring and support culture based on inclusivity and leader and peer-modelling.	✓	✓	✓	✓	Strong	12 weeks	5 x week	30 min	Afterschool program leader	Yes, 2 h pre-program with follow-up phone calls (x3) and emailed tips (x12)
Caldwell et al. (2022) [51] Canada	Afterschool program	*“The motivation*,* confidence*,* physical competence*,* knowledge*,* and understanding to value and take responsibility for engagement in physical activities for life”* International Physical Literacy Association (2014) [33]	BOKS Program: PA focussed: short movement bursts, movement-based games, and activities (2020)	X	X	X	N/a	Weak	∼ 12 weeks (one semester)	5 x week	NR (Mean duration delivered = 70 min/ week)	Afterschool program leader	Yes
Coyne et al. (2019) [50] Canada	PE	*“The motivation*,* confidence*,* physical competence*,* knowledge and understanding that individuals develop to maintain PA at an appropriate level throughout their life”* Whitehead (2010) [67]	RJTW program: progressive FMS skill development and consolidation through track and field inspired games, skill challenges in a non-competitive environment, focus on empowerment and personal development	✓	X	X	N/a	Moderate	10 weeks	2 x week	40 min	Specialist instructors	Yes (full- day training)
Farias et al. (2020) [54] Portugal	PE	*“The motivation*,* confidence*,* physical competence*,* knowledge and understanding to value and take responsibility for engagement in physical activities for life*” International Physical Literacy Association (2014) [33]	Year-long PE curriculum based on the Sport Education instructional model with action research elements (2013-14)	✓	✓	✓	N/a	Strong	1 school year	2 x week	45 min/ 90 min	PE teacher	Not mentioned
Invernizzi et al. (2019) [45] Italy	PE	*“An individual’s level of competence*,* autonomy and responsibility concerning physical and sports activities in lifelong participation in PA”* with reference to Whitehead (2010) [67]	PE program based on multi-teaching approaches (MTA) including reproductive and productive teaching styles which emphasize positive PA engagement and induce child’s autonomy	✓	✓	✓	✓	Strong	12 weeks	2 × 1 h PE session/week	60 min (approx.)	IV group: graduate PE students; Control group: class teacher	Yes; 3 × 2 h training session
Kriellaars et al. (2019) [49] Canada	PE	Multiple definitions discussed including Whitehead (2010) [67]	Altered focus of the skill development competency for PE (away from sport and small/medium sized games. Student-centred circus arts instruction in PE with student-centred instructional methods using competency progression checklists. (e.g., clowning, aerials, acrobatics, equilibriums, and manipulation)	✓	✓	✓	✓	Strong	∼ 12 weeks (one semester)	Mean: 3 x week (range 1–3)	mean duration of 56.6 min per class	Circus instructors Generalist/PE Teachers	Yes
Li et al. (2022) [53] Hong Kong	Classroom sit-stand desks, active recess play	None provided. Authors refer to Whitehead (2010) [67]. The theory of ecological dynamics is proposed as a framework to support the emergence of functional movement solutions to improve the quantity and quality of PA participation. Rudd et al. (2020) [68]	Blended PL intervention combining sit-stand desks into classrooms and play-based recess activities led by PE interns (e.g. skipping rope, shuttlecock kicking, hide-and-seek) (2019)	✓	✓	✓	N/a	Strong	13 weeks	Standing breaks every 15-mins during 2 classes; Active recess (up to 15-mins, twice per day across the week)	Min 1 h standing p/day; Active recess 15 min	Teacher (classroom) PE intern (recess)	Yes, 3 h briefing session
Liu and Chen (2022) [56] USA	PE	“*The motivation*,* confidence*,* physical competence*,* knowledge and understanding to value and take responsibility for engagement in physical activities for life.”* Whitehead (2010) [67]	SDT-guided pedagogical workshops, with a long-term vision on PYD. Content and delivery based on Heart PL model; children learn in high-low PL performing dyads (2019)	✓	✓	✓	N/a	Strong	8-weeks	2 x week	20–30 min	Research assistant	Not mentioned
Mandigo et al. (2019) [48] Canada	Afterschool program	*“The motivation*,* confidence*,* physical competence*,* knowledge*,* and understanding to value and take responsibility for engagement in physical activities for life”* International Physical Literacy Association (2014) [33]	TgfU lessons designed using the PlaySport program, based on principles of sampling, representation, exaggeration, and tactical complexity	✓	✓	✓	N/a	Strong	8-weeks	∼ 3 x week (25 sessions in total)	∼ 60 min	Mostly under-graduate PE students. Some by others (e.g., developer of the lesson plans, teacher, lead author)	Yes
Mendoza-Muñoz et al. (2022) [55] Spain	Structured recess	*“The motivation*,* confidence*,* physical competence*,* knowledge and understanding to value and participate in a physically active lifestyle”* ICSSPE UNESCO de Balazs et al. (2017) [69]	Recreational (recess-based) AB program based on games, which aimed to improve motivation and motor skills in schoolchildren	✓	✓	X	N/a	Moderate	4-weeks	Daily	15-mins	Specialist instructors	N/a
Stoddart et al. (2021) [47] Canada	PE	*“The motivation*,* confidence*,* physical competence*,* knowledge*,* and understanding to value and take responsibility for engagement in physical activities for life”* International Physical Literacy Association (2014) [33]	PlitPE intervention was informed by SDT and built around two principles of learning: (a) repetition of skills and (b) knowledge of results, that included augmented feedback and positive challenges (2015)	✓	✓	✓	N/a	Strong	8–9 weeks	3 x week	25 min within a 50 min PE lesson	PE or class teacher supported by mentor PE teacher (writing lesson plans, teaching games)	Yes (embedded professional development model)
Telford et al. (2021) [52] Australia	PE; active recess; classroom activity breaks; community engagement	*“The motivation*,* confidence*,* physical competence*,* knowledge*,* and understanding to value and take responsibility for engaging in physical activities for life”* Whitehead (2010) [67]	Multi-component PEPL intervention that aimed to (a) improve the school climate of PL; (b) improving delivery and frequency of PE; (c) promote schoolyard PA; (d) create links between the school & community-based organisations	✓	✓	✓	✓	Strong	33 weeks	Different activities, including one additional PE lesson/week and four activity sessions (15–40 min) in the school yard/ week		PEPL coach to train and mentor teachers	Yes

For the PL domains of learning targeted ‘✓’ means the study described how the intervention targeted the domain identified in the PL definition/ philosophical approach; ‘X’ the study did not describe how the intervention targeted the domain identified in the PL definition/ philosophical approach; ‘N/a’ the PL definition and intervention content did not include this domain. For alignment between PL definition/ approach & intervention content: Strong = Good alignment between intervention content and PL definition/approach. An explanation was provided for how intervention supports PL development across each of the domains identified in the PL definition/approach; Moderate = Some alignment between intervention content and PL definition/approach but not all domains explained; Weak = Little or no alignment between intervention content and PL definition/approach. PL may not be defined, or intervention aims and content in the context of physical literacy is not described. Abbreviations: AB = active breaks; Active recess = optional physical activity opportunities during recess; BOKS = Build Our Kids’ Success; Cogn = cognitive domain; FMS = fundamental movement skills; ICSSPE UNESCO = International Council of Sport Science and Physical Education of the United Nations Educational, Scientific and Cultural Organization; IPLA = International Physical Literacy Association; ; IV = Intervention; MTA = Multiple Teaching Approach; mins = minutes; NR = not reported; mins = minutes; PA = physical activity; PE = physical education; PEPL = Physical education and physical literacy approach; Phys = physical domain; PL = physical literacy; PlitPE = physical literacy enriched physical education; PYD = positive youth development; Psych = psychological domain; RJTW Program = run, jump, throw, wheel program; SDT = social determination theory; Soc = social domain; TgfU = teaching games for understanding; ^a^ = The ‘PL domains of learning targeted’ is based on the description of intervention content and pedagogical approaches used in each study. There is divergence internationally over whether PA is a domain of PL. In this Table, we have not included PA as a domain of PL, but all interventions were grounded in movement and involved physically active lessons, games, activities and/or play, indicating some alignment between intervention content and the PL definitions that include PA as a domain

Five studies reported the year the intervention was conducted. Of these, three were in the last five years [51, 53, 56], and two were 10 years or more ago [47, 54]. Of the two older interventions, both demonstrated strong alignment between adopted definition (both IPLA) and intervention content, with one using an established physical literacy assessment tool [47], and the other qualitative assessment methods [54].

Interventions also varied in delivery characteristics. With respect to length, five interventions were less than a school term (4–10 weeks) [47, 48, 50, 55, 56], five were approximately one term (∼ 12 weeks) [45, 46, 49, 51, 53], and two, an entire school year [52, 54]. Most were delivered two to three times per week for a duration of 20 to 60 min per session. For multi-component interventions, there were several activities throughout the school day, such as standing or activity breaks during class time, PE classes, and structured recess activities and games [53], or throughout the school week [52]. Two thirds (*n* = 8) of interventions were delivered by the PE or classroom teacher, or by the regular program leader (for afterschool programs). In most interventions (*n* = 10) provider training was offered, with only two studies not offering (or describing) the provider training component of their intervention [54, 56].

### Risk of Bias

Risk of bias across the quantitative and qualitative domains for studies is shown in Table S6 of the ESM. In relation to quantitative assessment of 10 studies, all used validated measures of physical literacy. Other risk of bias aspects were reported in more than half of the studies, i.e., seven described dropout, seven had ≤ 20% for < 6-m follow-up or ≤ 30% for ≥ 6-m follow-up, and seven accounted for potential confounders. In comparison, five had comparable baseline characteristics between groups, three had outcome assessors blinded, three reported sample size/power calculation and adequately powered their study, and three used an intention to treat analysis. Only one study clearly described and carried out the randomisation process and only one followed participants for ≥ 6-m.

In relation to qualitative assessment of five studies, all considered ethical issues. A clear statement of aims, appropriate qualitative methodology and an appropriate recruitment strategy was reported in a total of three studies for each aspect. Two studies reported designs appropriate to the aims, collected data in a way that addressed the research aims, and had a clear statement of findings. Only one study had clear and consistent theoretical underpinnings, and only one study described sufficiently rigorous data analysis.

### Assessment of Physical Literacy and Other Outcomes

For quantitative results, instruments used to assess physical literacy and other outcomes including PA are described below and mapped by study and physical literacy domain and/or element or additional outcome in Table S7 of the ESM. Qualitative methodology for studies that collected data on perceived physical literacy outcomes is provided in Table S8 of the ESM.

#### Physical Literacy Assessment

Assessment of physical literacy was performed using instruments specifically designed to assess children’s physical literacy outcomes across various domains and/or elements *and* combinations of validated or novel measures to assess each domain and/or physical literacy element of interest separately. Seven interventions used the Canadian-developed physical literacy assessment instruments, namely the Physical Literacy Assessment for Youth (PLAY) suite of instruments (PLAY*fun*, PLAY*self*, PLAY*inventory*) [46, 47, 49] or the Canadian Physical Literacy Assessment instruments (CAPL-1, CAPL-2, CAPL-2 Chinese, validated for use in Chinese populations) [50, 53, 55, 56]. Although the PLAY*self* questionnaire assesses the physical, affective, and cognitive domains of physical literacy, outcomes are not reported at the individual domain level. In contrast, CAPL provides a total physical literacy score, in addition to reporting results by physical literacy domain and/or element. Another Canadian tool, Passport for Life (PFL) [48], was used in one study, and reported results across five physical literacy domains: physical, affective, cognitive, social, and behavioural, but did not provide an aggregate physical literacy score. This was the only instrument that measured the social domain.

Two interventions utilised their own combinations of validated and novel measures and scales to assess physical literacy domains and/or elements individually [45, 52]. Both used the Test of Gross Motor Development 2 (TMGD-2) to assess children’s motor competence, but Invernizzi et al. [45] also included the multi-stage fitness test (MFT) to assess cardiovascular endurance, whereas this element was not assessed by Telford et al. [52]. Likewise, both studies used variations of the Physical Activity Enjoyment Scale (PACES) to assess children’s enjoyment of PA for the affective domain. These studies did not provide an overall physical literacy score.

#### Other Outcomes

All but one study measured PA [46]. Seven studies measured PA as a behavioural domain of physical literacy [47–50, 53, 55, 56] and two studies measured PA as an additional outcome [45, 52]. Across studies, PA was measured using device-based assessments (accelerometer, heart rate monitor, pedometer) and by self-report (CAPL-1, CAPL-2, CAPL-2 (Chinese), PLAY*inventory*, PFL, PA questionnaire-Children, single item PA scale). Only one study assessed outcomes beyond PA or their conceptualisation of physical literacy. Li et al. [53] measured different aspects of cognitive performance using computer-based tools (i.e., Wisconsin Card Sorting Test and Tower of London task).

#### Qualitative Data Collection Methodology

Five studies collected data qualitatively to examine the influence of school-based interventions on children’s physical literacy development. Four were mixed methods, combining quantitative assessments with interviews or focus groups [45, 51, 52, 56]. The fifth study used a multi-method qualitative enquiry in the form of a retrospective examination of physical literacy development of seventh grade children who participated in a year-long Sport Education curriculum in Portugal [54]. The latter combined augmented memory retrieval techniques and a retrospective survey with informal semi-structured individual and focus group interviews.

### Intervention Outcomes

Across studies, there was substantial variability in how physical literacy domains and elements were conceptualised and measured, the number of tests performed per domain/element, and how results were reported. For example, for object control skills, one study measured throwing and kicking [PFL movement skills test) [48]], while two others combined striking, bouncing, kicking, under and overhand throwing, and catching (TGMD-2) [45, 52]. However, these two studies reported the TGMD-2 results differently, with one providing a composite motor competence score (combining object control and locomotor) [45], and the other reporting object control and locomotor scores separately [52] (see Tables 3 and 4). We discuss these results below by physical literacy domain, PA (sometimes assessed as a behavioural domain) and other outcomes. Throughout, we integrate the qualitative findings (see Table 5) with the quantitative test results (see Tables 3 and 4) to provide deeper insight and understanding of how school-based interventions develop children’s physical literacy. Tables 3 and 4 identify the school context (i.e., PE, structured recess, after school or multi-component) for each intervention, but there were not enough studies to meaningfully identify any patterns for intervention effects based on school context.

**Table 3:** Controlled designs - intervention effects by physical literacy domain, element, or other outcome

PL Domain and/or element mapped to APLF^a^ / Other outcome	Description in paper (if different to APLF or multiple test results per element/item)	Bremer et al. (2020) [46]	Invernizzi et al. (2019) [45]	Kriellaars et al. (2019) [49]	Stoddart et al. (2021) [47]	Mendoza-Muñoz et al. (2022) [55] (Structured recess)	Li et al. (2022) [53]		Telford et al. (2021) [52]	Summary (Sig. result in expected direction)	Evidence level for total and each domain
		(After school)	(PE)	(PE)	(PE)		(Multi-component)		(Multi-component)		
							IG1:SS-Play	IG2: Play			
**Total PL**										**2/4**	**?**
	Self-assessment of PL (physical, affective, cognitive domains)	0		+	0					1/3	
Composite score: Physical, affective, cognitive, behavioural (PA) domains						+				1/1	
**Physical Domain**										**10/15**	**++**
Composite score: agility, cardiovascular endurance, coordination, movement skills, muscular endurance, object manipulation, stability/balance	Physical competence: cardiovascular and musculoskeletal fitness, motor competence					+	Pre-Post: + Pre-Follow up: 0	Pre-Post: + Pre-Follow up: 0		3/5	
Composite score: movement skills, object manipulation, stability/balance	Running, locomotor, object control-upper body, object control-lower body, balance	0		+	+					2/3	
Composite score: movement skills, object manipulation	Locomotor skills, object control		+			+				2/2	
Cardiovascular endurance			+			0				1/2	
Muscular endurance						+				1/1	
Object manipulation	Object control								+	1/1	
Movement skills	Locomotor skills								0	0/1	
**Affective Domain**										**9/22**	**?**
Composite score: motivation, confidence, engagement and enjoyment, self-perception	Motivation, self-competence, predilection, adequacy					+	Pre-Post: 0 Pre-Follow up: 0	Pre-Post: 0 Pre-Follow up: 0		1/5	
Motivation		0				+				1/2	
Confidence	Self-efficacy / self-competence	0				+				3/6	
	Relation-inferred self-efficacy (Leader score) Relation-inferred self-efficacy (Peer score)	+ 0									
	Other-efficacy (Leader score) Other-efficacy (Peer score)	+ 0									
Enjoyment & engagement	PA enjoyment	+	+						0	3/4	
	Predilection					+					
Self-perception	Self-concept (academic, non-academic, global		0							1/5	
	Adequacy					+					
	Sports competence Physical condition Physical self-worth								− 0 0		
**Cognitive Domain**										**1/5**	**0**
Content knowledge	Knowledge and understanding					+	Pre-Post: 0 Pre-Follow up: 0	Pre-Post: 0 Pre-Follow up: 0		1/5	
**Social Domain**											
N/A											
**Other Outcomes – Physical Activity (Behavioural Domain)**										**5/26**	**0**
Amount of PA	Composite score: daily self-reported PA, pedometer					0	Pre-Post: + Pre-Follow up: 0	Pre-Post: + Pre-Follow up: 0		2/5	
	Daily self-report PA					+				1/1	
	Weekly self-reported PA		+							1/1	
Amount and/or intensity of PA (device measured)	Daily step counts					0				0/1	
	Daily LPA						Pre-Post: 0 Pre-Follow up: 0	Pre-Post: 0 Pre-Follow up: 0		0/4	
	Daily MPA						Pre-Post: 0 Pre-Follow up: 0	Pre-Post: 0 Pre-Follow up: 0		0/4	
	Daily VPA						Pre-Post: 0 Pre-Follow up: 0	Pre-Post: 0 Pre-Follow up: 0		0/4	
	Daily MVPA Daily PA School MVPA School PA								0 0 0 0	0/4	
Participation in activities	Number of physically active pursuits			+	0					1/2	
**Other Outcomes – Cognitive performance**										**2/8**	**0**
	Cognitive flexibility						Pre-Post: 0 Pre-Follow up: +	Pre-Post: 0 Pre-Follow up: 0		1/4	
	Planning						Pre-Post: 0 Pre-Follow up: 0	Pre-Post: 0 Pre-Follow up: +		1/4	

Intervention effects for each study are mapped to the APLF, a behavioural domain of physical literacy i.e., PA, or a wider area of interest i.e., cognitive performance. All physical activity outcomes are reported under the ‘behavioural domain’ for ease of reading (see Supplementary file 3 for explanation of the PL instruments and measures utilised across studies). One study (Mendoza-Muñoz et al. [55]) reported test outcomes for both individual items *and* the sum of items for each PL domain. (^a^) Mapping of physical literacy instruments to the Australian Physical literacy Framework derived from systematic review by Barnett et al. [58]. SYMBOLS: (**+**) significant improvements in the expected direction in the IG compared against CG (*p* < 0.5) pre-to post intervention; (**0**) No difference between IG & CG pre to post intervention; (**−**) decline in the IG pre to post intervention or improvement in CG but not IG. Coding for ‘Evidence Level’ adapted from Lubans et al. [36] and Page et al. [37]: PL domains were given a ‘0’ where less than 35% of tests found a significant improvement; ‘?’ where 35–59% of tests found an improvement; ‘+’ where 60–100% of tests found an improvement; and ‘++’ where 60–100% of tests found an improvement (≥ 4 studies). There was no denominator or minimum number of studies in this coding scheme, meaning a stronger evidence level could be reported for domains less examined, if positive results in those tests were reported. *Abbreviations* APLF = Australian Physical literacy Framework; CG = control group; IG = intervention group; LPA = light physical activity; MPA = moderate physical activity; MVPA = moderate to vigorous physical activity; PA = physical activity; PE = physical education; PL = physical literacy; SSPlay = Sit-Stand Play; VPA = vigorous physical activity

**Table 4:** Uncontrolled designs - intervention effects by physical literacy domain, element, or other outcome

PL Domain and/or element mapped to APLF^a^ /	Description in paper (if different to APLF or multiple test results per element/item)	Mandigo et al. (2019) [48]	Coyne et al. (2019) [50]	Liu and Chen (2022) [56]		Summary (Sig. result in expected direction)	Evidence level for total and each domain
Other outcome		(After-school)	(PE)	(PE)			
				High PL Group	Low PL Group		
**Total PL**						**2/3**	**+**
Composite score: Physical, affective, cognitive, and behavioural (PA) domains			+	− ^MedES^	+ ^MedES^	2/3	
**Physical Domain**						**4/9**	**?**
Composite score: agility, cardiovascular endurance, coordination, movement skills, muscular endurance, object manipulation, stability/balance	Physical competence: cardiovascular and musculoskeletal fitness, motor competence		+	− ^SmES^	+ ^SmES^	2/3	
Composite score: movement skills, coordination		0				0/1	
Cardiovascular endurance		+				1/1	
Muscular endurance		0				0/1	
Object manipulation	Throwing and catching Kicking	0 0				0/2	
Stability/balance		+				1/1	
**Affective Domain**						**2/4**	**?**
Composite score: motivation, confidence, engagement and enjoyment, self-perception	Motivation, self-competence, predilection, adequacy		0	− ^SmES^	+ ^SmES^	1/3	
Composite score: motivation, confidence, engagement, self-perception, self-regulation-emotional, self-regulation-physical	Feeling: engagement, motivation	+				1/1	
**Cognitive Domain**						**3/4**	**+**
Content knowledge	Knowledge and understanding		+	+ ^SmES^	+ ^SmES^	3/3	
Composite score: knowledge, reasoning, strategy and planning	Thinking: Knowledge, understanding, critical thinking, goal setting	0				0/1	
**Social Domain**						**0/1**	**0**
Composite score for collaboration, ethics, relationships, society, and culture	Interacting: problem-solving, personal management, cooperation, social skills	0				0/1	
**Other Outcomes – Physical Activity (Behavioural Domain)**						**3/9**	**0**
Amount of PA	Composite score: daily self-reported PA, pedometer		0	− ^MedES^	+ ^SmES^	1/3	
Amount and intensity of PA (device measured)	PE LPA PE MPA PE VPA		0 − 0			0/3	
Participation in activities	Diverse activities Diverse environments Diverse interests	0 + +				2/3	

Intervention effects for each study are mapped to the APLF or a behavioural domain of physical literacy i.e., PA. All physical activity outcomes are reported under the ‘behavioural domain’ for ease of reading (see Supplementary file 3 for explanation of the PL instruments and measures utilised across studies). (^a^) Mapping of physical literacy instruments to the Australian Physical literacy Framework derived from systematic review by Barnett et al. [58]. SYMBOLS: (**+**) significant improvements in the expected direction within group pre-to post intervention (*p* < 0.5); (**0**) No change within group pre to post intervention; (**−**) decline within group pre to post intervention. Coding for ‘Evidence Level’ adapted from Lubans et al. [36] and Page et al. [37]: PL domains were given a ‘0’ where less than 35% of tests found a significant improvement; ‘?’ where 35–59% of tests found an improvement; ‘+’ where 60–100% of tests found an improvement; and ‘++’ where 60–100% of tests found an improvement (≥ 4 studies). There was no denominator or minimum number of studies in this coding scheme, meaning a stronger evidence level could be reported for domains less examined, if positive results in those tests were reported. Abbreviations: APLF = Australian Physical literacy Framework; LPA = light physical activity; MedES = medium effect size (Hedges’ g ≥ 0.5 or Eta squared 0.06); MPA = moderate physical activity; PA = physical activity; PE = physical education; PL = physical literacy; SmES = small effect size (Hedges’ g ≥ 0.2 or Eta Squared 0.01); VPA = vigorous physical activity

**Table 5:** Themes for intervention effects synthesised from qualitative findings

Physical literacy domains of learning APLF Elements/ other outcomes	Theme	Caldwell et al. (2022) [51]	Farias et al. (2020) [54]	Invernizzi et al. (2019) [45]	Liu and Chen (2022) [56]	Telford et al. (2021) [52]
Physical domain						
Movement skills	Skilful motor control including hand-eye coordination		✓		✓	✓
Muscular endurance	Increased muscular endurance	✓			✓	
Strength	Increased muscular strength	✓			✓	
**Affective domain**						
Confidence	Confidence in ability, willingness to try new things	✓	✓		✓	✓
Engagement	Willingness to exercise, enthusiasm to participate in PA	✓	✓		✓	✓
	Commitment to equitable and meaningful PE		✓			
Enjoyment	Happier	✓		✓		
	Enjoyment in activities, learning new things			✓	✓	✓
Motivation	More motivated to participate in PE activities		✓		✓	✓
	Increased desire to play outside					✓
Self-perception	Improved self-worth		✓		✓	✓
Self-regulation (emotions)	Resilience to discriminatory practices e.g., sex-based Less reactive to stress/conflict	✓	✓			
Self-regulation (physical)	Improved energy and mood	✓				
**Cognitive domain**						
Content knowledge	Appreciate importance of fitness	✓				
Reasoning	Better at accepting and dealing with criticism		✓	✓		
	Valuing improvement over winning		✓			✓
Rules	Able to create and/or set-up and run their own games		✓	✓		✓
Tactics, strategy, planning	Tactical understanding & problem solving		✓		✓	✓
**Social domain**						
Collaboration	Collaboration and teamwork		✓		✓	✓
	Less conflict in class	✓				
	Developed leadership		✓			
	Able to re-teach family and others					✓
Ethics	More peer-to-peer accountability e.g., learned to play nicely		✓			✓
Relationships	Empathy towards others, generation of positive social attitudes		✓			
	Learned to read nonverbal communication					✓
	Interacted more with classmates		✓			✓
Society & culture	Created ‘healthy’ competition within class		✓			
**Other outcomes**						
**Physical activity** (Behavioural domain)	Increased PA and more/different types of PA				✓	
	Attended ‘extra’ sessions at school					✓
	Engaged in more community sport					✓
	Appreciated chance to just play	✓				
	Improved compliance and behavioural standards	✓		✓		✓
**Cognitive performance**	Improved concentration and focus	✓				✓

Table details the themes synthesised from qualitative findings for the intervention effects. Themes are mapped by study to the APLF, a behavioural domain of PL (i.e., PA), or other outcomes (i.e., cognitive performance). Abbreviations: APLF = Australian Physical literacy Framework; PA = physical activity; PE = physical education

#### Total Physical Literacy

Six interventions reported a total physical literacy score [46, 47, 49, 50, 55, 56]. For the controlled designs, results were mixed, with significant results in the expected direction for 2 of 4 tests based on a composite physical literacy score (combining objective and self-report measures) using CAPL-2 [55] or self-assessment of physical literacy using the PLAY*self* questionnaire [49]. For the uncontrolled designs, significant results in the expected direction were reported for 2 of 3 tests, both of which provided a composite physical literacy score using the CAPL-1&2 tools [50, 56]. The small within subjects study by Liu and Chen [56] divided participants into two groups [i.e. (i) high and (ii) low-performing physical literacy at baseline], and reported significant improvements for the low-performing group (medium effect size) but a significant decline in the high-performing group at the end of the eight-week PE intervention.

#### Physical Domain

All studies assessed constructs mapped to the physical domain of physical literacy, and all used objective methods to measure either single elements or various combinations of elements. For controlled designs, two thirds of the tests (10 of 15) reported significant results in the expected direction, indicating positive evidence for an effect. This effect was commonly observed in tests that measured several physical elements (e.g., movement skills, object manipulation, stability/balance) and provided a composite score. The results were less consistent when individual physical elements were measured e.g., movement skills or cardiovascular endurance. For uncontrolled designs, there was mixed evidence as less than half of the tests (4 of 9) performed reported a significant result in the expected direction.

Consistent with quantitative outcomes for the controlled studies, qualitative findings for the physical domain were positive, with children in the year-long Australian physical education and physical literacy (PEPL) intervention describing via focus groups their improved hand-eye co-ordination and becoming more skilful at certain movements, which were supported through objective assessment for object control, but not locomotor skills [52]. Additionally, Build Our Kids’ Success (BOKS) program leaders (a Canadian afterschool program) perceived marked increases in children’s muscular strength and endurance (e.g., number of push-ups performed) [46]. The Portuguese case study evaluating a year-long Sports Education program reported rich qualitative findings, with many students perceiving they had increased their movement capabilities through ‘the project’ by developing a “*strong basis of game-play skills and understanding of sport*” [54, p.271]. Children in this intervention described many factors that contributed to their enhanced physical skills, including the autonomy and ownership of decision making about the content and pacing of the learning activities, as well as the peer-teaching and cooperative dynamics (debates-of-ideas) embedded in the curriculum [54].

#### Affective Domain

Across the physical literacy domains, the highest number of tests were mapped to constructs in the affective domain, and all used self-report or proxy-report methods. This was also the domain with the most variability in how constructs were defined and assessed. For example, variations of the element ‘confidence’ were assessed in five different ways, as were variations of the element ‘self-perception’. For controlled designs, less than half the tests (9 of 22) reported significant results in the expected direction. Significant results were more commonly reported for single elements [e.g., engagement and enjoyment (3 of 4 tests), confidence (3 of 6 tests)] than for tests that measured several affective elements together. For uncontrolled designs, only tests that measured several elements and provided a composite score were performed, with mixed evidence: 50% (2 of 4) reported a significant result in the expected direction.

In contrast with the quantitative outcomes, qualitative findings were positive across all five studies, indicating perceived improvements in children’s ‘confidence’, ‘motivation’, ‘self-perception’, and ‘enjoyment’. Children participating in the PEPL program described becoming “*more motivated to participate in PE activities*”, “[having] *enjoyed continuing these activities and games in school recess times*”, and “*developed more confidence to ‘have a go’ at new activities without fear of failure*” [52, p.102]. Program leaders in the BOKS program also observed improvements in children’s willingness to participate in PA and students reported building the confidence to try new activities and make new friends [51]. Likewise, children in the Sport Education program reported high enthusiasm and engagement in the learning activities, and improved motivation, confidence and self-perception to extend sport participation both inside and outside the school context [54]. As one child explained: “*I was a poor volleyball player in the sixth-grade*,* but ‘the project’ made me realise I did have awesome skills. I teamed-up with Duarte* [other child] *and we won the 2v2 tournament*” [54, p.271]. Children in the American positive youth development-focussed PE workshop intervention described improved enjoyment and motivation to be active [56]. Children participating in an Italian PE intervention based on multi-teaching approaches described satisfaction and enjoyment in learning new abilities, as one child described: “*the most important thing is to have fun. Physical activity should be a pleasant moment*,* and it was so*” [45, p.8].

#### Cognitive Domain

There were fewer tests performed for cognitive aspects of physical literacy. For controlled designs, only the element ‘content knowledge’ was assessed, and this did not meet our threshold for positive intervention effects with only 1 of 5 tests reporting a significant result in the expected direction. Whereas for uncontrolled studies, 100% of tests (3 of 3) assessing ‘content knowledge’ reported significant improvements. Additionally, one test assessed several elements (knowledge, reasoning, strategy, and planning) but did not report a significant improvement. Overall, 3 of 4 tests showed results in the expected direction, providing positive evidence for this domain.

Qualitative findings for the cognitive domain indicated positive results across a wider range of physical literacy elements than was measured quantitatively. In addition to improved ‘content knowledge’ [26, 45, 51, 54], findings suggested an improvement in children’s understanding of ‘rules’ [45, 52, 54], ‘tactics’ [52, 54], ‘reasoning’ [45, 52, 54] and ‘strategy and planning’ [54, 56]. A child in the Sport Education program described how these cognitive capabilities evolved: “*The skills I developed in the seventh grade expanded my performance of basketball*,* particularly my off-the ball game. First*,* it was all about getting the ball myself. Then I became more concerned about opening space*,* helping my teammates reach a higher level and score more*” [54, p.272].

#### Social Domain

Only one study assessed the social domain quantitively [48]. Using the self-report PFL tool [57] that assessed several social elements (collaboration, ethics, relationships, society, and culture), this uncontrolled study reported no change pre- to post-intervention.

Qualitative findings for the social domain indicated positive effects from four studies for several elements, including ‘collaboration’, ‘ethics’ and ‘relationships’. Students participating in the PEPL program reported improved teamwork and collaboration, describing how they learned to negotiate rules and create games together [52]. Children also described how their relationships with their peers improved, as one child explained: *“Before we did this I never used to talk to people in my class. I stuck to people in my group*,* like my friends. But now…like…I talk to other people. Before I used to be shy*,* but not anymore”* [52, p.103]. Likewise, students participating in Sport Education program indicated several interpersonal skills were developed, including an enhanced sense of fairness, reciprocity, and sensitivity to others, which led them to be more inclusive, cooperative and empathetic in their social interactions [54]. As one teacher in this study observed: “*All the other class teachers saw outstanding groupwork skills in them. They were bonded to each other like no other class I ever had*,* and I believe this was a consequence of the augmented cooperation and teamwork promoted in the seventh-grade PE*” [54, p.273]. A child described her experience in the project as ‘life-changing’: “*In the sixth grade*,* I was an outsider in the class. Then ‘the project’ changed my life. Everyone invested so much in helping me improve at different levels*,* so I felt compelled to change the way I interacted with people. I felt I should return this caring to other teammates too*” [54, p.272].

#### Other Outcomes - Physical Activity

PA was the most measured construct across studies, either as a behavioural domain of physical literacy, or as an additional outcome of interest. Several studies measured PA in multiple ways, which increased the total number of tests performed. For controlled designs, only 5 of 26 tests reported a significant improvement in the expected direction, and these were all self-report assessment tools, bar one composite test which was made up of self-report and pedometer step counts. The tests using device-based methods alone did not yield significant improvements in any study. For uncontrolled designs, only one-third of tests (3 of 9) reported significant improvements. There was also a similar pattern to the controlled studies of more positive results in tests using self-report assessment tools.

In contrast, increases in PA participation were described in all five studies that assessed intervention outcomes qualitatively, both during school hours [51, 54] and outside of school [45, 52, 54, 56]. For example, a child reflecting on the Sport Education program described how the enthusiasm to participate in activities acquired in the seventh grade carried forward into subsequent years: “*There was this volleyball school tournament in which none of the other girls in the class wanted to participate. But we wanted to play so much that we mobilized the entire class. Even those girls who didn’t like PE*” [54, p.273]. Likewise, children participating in the PEPL program described “*increased involvement in community sport*” and “*choosing physical activity in* [their] *free time*” [52, p.104].

#### Other Outcomes - Cognitive Performance

The one intervention that assessed outcomes beyond physical literacy or PA, measured cognitive flexibility and cognitive planning in two intervention arms and at two time points [53]. Only 2 of 8 tests in this study reported a significant result in the expected direction, one for each intervention group at follow-up, but in different tests: cognitive flexibility improved for the ‘sit-stand and active play’ group while planning improved for the ‘active play only’ group [53].

## Discussion

This review examined the efficacy of school-based interventions that sought to increase children’s physical literacy and whether these physical literacy interventions impacted other outcomes. To do this, we synthesised data from 12 interventions that adopted a holistic, interconnected conceptualisation of physical literacy, as represented by the APLF [4]. Our findings from quantitative data indicate mixed evidence for holistic physical literacy interventions improving total physical literacy, positive evidence for improving the physical components, mixed evidence for improving the affective and cognitive domains, and no support for improving the social domain. Notably domains were not all evaluated equally, with only one study investigating the social domain. Qualitative findings indicated perceived benefits across all domains, including those less frequently examined in quantitative assessments.

We found mixed evidence for holistic physical literacy interventions improving total physical literacy. Only four studies examined total physical literacy, with 57% (4 of 7 tests) in the expected direction. One recent systematic review by Liu and Chen [26], which identified 20 child and youth physical literacy interventions (one study [48] was also assessed in the current review), also reported mixed findings with no clear conclusions, although studies included in that review spanned a wider age range (1–15 years) and several settings including early childhood centres, schools and community settings. Additionally, that review included articles which drew on the concept of physical literacy *or* had physical literacy as an outcome, and therefore some studies assessed a narrow conceptualisation of physical literacy that was limited to the physical domain [26]. The 2022 review by Carl et al. [15] on physical literacy interventions (conducted with adults and children across settings, *n* = 48 studies) included five studies which assessed total physical literacy, and more than half of these (three) showed significant treatment effects (moderate to high), although study heterogeneity was substantial. Only one study, with no effect [46], was also assessed in the current review. Like Liu and Chen [26], Carl et al. [15] included articles which drew on the concept of physical literacy *or* had physical literacy as an outcome, and therefore some included studies also assessed a narrow conceptualisation of physical literacy that was limited to the physical domain. Additionally, Carl et al. [15] conducted a meta-analysis with the controlled designs only, whereas we expanded this and have reported findings for both controlled and uncontrolled designs. Other recent reviews in child and youth populations cannot be easily compared to our results. For example, a 2021 scoping review had a wide inclusion criterion regarding the intervention topic (any strategy that aimed to improve PA *or* any component of physical literacy) but a narrow focus in relation to the population (aged 6 to 18 years enrolled in a Brazilian school) [25].

For the physical domain overall, we found mixed evidence of a positive effect with 58% (14 of 24 tests) in the expected direction. However, there was strong positive evidence (67%) for the controlled studies (10/15) rather than uncontrolled designs (4/9). Qualitative findings for the physical domain supported the positive quantitative results. Our findings for the physical domain are consistent with the recent review and meta-analyses by Carl et al. [15], which reported a significant impact (large effect size overall) on indicators of physical competence. In that review, sensitivity analysis indicated effect size differences could not be attributed to trial design (in terms of randomization) but those authors did not conduct a sensitivity analysis by study design. Also, the recent review by Anico et al. [28] on the efficacy of school-based run/walk programmes for children and adolescents to develop physical literacy and PA reported that their included 10 interventions (no overlap with the studies in the current review) contributed to improved physical performance. Even though the review by Anico et al. [28] did not include interventions with a specific focus on physical literacy, their findings provide support for PA interventions positively impacting on physical literacy.

For the affective and cognitive domains, we found mixed evidence from eight studies. In the affective domain, a wide number of elements were assessed, with 42% of tests in the expected direction. In contrast, qualitative findings for the affective domain were positive across all studies. Similarly, for the cognitive domain, we found mixed evidence from five studies, with 44% in the expected direction. Qualitative findings for the cognitive domain supported these positive results but were across a wider range of factors than those measured quantitatively. The review by Carl et al. [15] examined ‘motivation and confidence’ as a domain (including enjoyment and positive effect) and reported a significant positive effect, although the authors also noted mixed findings, i.e., when motivation was examined on its own, nearly all studies (9 of 10) did not find an effect. Carl et al. [15] also reported a significant medium-size effect for their investigated domain of ‘knowledge and understanding’, although study heterogeneity was present. The review on the efficacy of school-based run/walk programmes for children and adolescents also reported that some aspects of the affective domain (motivation and emotional regulation) improved [28].

We found no evidence for the social domain as only one uncontrolled study assessed the social domain quantitively and reported no change pre- to post-intervention [48]. The review by Carl et al. [15] did not investigate this domain of physical literacy as this did not align with their definition. The lack of assessment of the social domain in the quantitative data is likely because most definitions of physical literacy do not include the social domain, and therefore, fewer instruments measure this domain [58, 59]. Positive outcomes for a range of elements in the social domain were reported in the qualitative data, providing direction for the design of future interventions and assessment instruments.

Generally, our results are consistent with other physical literacy related reviews for the physical domain in that there were more effects on outcomes within the physical domain [15, 28]. Given five of the interventions in the current review were PE based, it is of note that a large meta-analysis of learning interventions (*n* = 135) within PE also reported the greatest effects were within psychomotor learning outcomes (*d* = 0.52), compared to affective (*d* = 0.47), social (*d* = 0.32), and cognitive (*d* = 0.17) outcomes [60]. In the current review, all studies that measured quantitative outcomes assessed constructs mapped to the physical domain of physical literacy with the other domains less investigated. Carl et al. [15] also reported that while most interventions listed at least one outcome in the physical domain, fewer studies included assessments related to the affective and the cognitive domains. The review by Anico et al. [28] also reported a lack of holistic scope in that none of their included studies investigated all three physical literacy domains (as they were defined) or the cognitive domain.

For the other domains/elements of physical literacy there are mixed findings between our review and previous reviews, likely attributable to the different definitions of physical literacy applied and the scope of previous reviews. Uniquely, our review is the first to synthesise evidence for holistic physical literacy interventions conducted with children aged 5–14 years at school. Studies in the current review varied in how the intervention content aligned with a multidimensional understanding of physical literacy, with nine of the 12 interventions demonstrating strong alignment between their adopted definition, the intervention or program and the outcomes evaluated. Carl et al. [15] acknowledged that an insufficient number of the interventions in their review were evaluated holistically, despite adopting a ‘holistic’ definition and suggested that future studies could more specifically link physical literacy theory to the intervention across the whole process, i.e., from conceptualisation and definition to the objectives and content to the final evaluation. The mixed findings and small number of studies included in the current review reinforce this recommendation, which will help to drive the field forwards and ensure a more transparent and realistic understanding of intervention efficacy.

Finally, there was no evidence for interventions impacting other outcomes. One study measured cognitive performance but did not report intervention effects. PA was the most measured construct (quantitatively) across nine studies in the current review yet only 28% of tests reported that physical literacy interventions improved PA. In contrast, the review by Carl et al. [15] reported that 11 interventions used PA assessments after the intervention and apart from one study, the mean values all favoured the intervention groups (low-to-moderate effect size). Their findings are likely different to ours for several reasons: a different physical literacy definition and wider scope with respect to population group and setting was used, which meant there were only three studies in common for PA, and the different methodology that meant not all tests conducted in each study were included in their review. Interestingly our findings highlight that even though the physical components of physical literacy improved, there were no corresponding improvements in PA. This goes against the theoretical premise of physical literacy that improvements in physical literacy components will positively impact PA. There is likely a time factor involved, as it is logical that the development of the physical elements of physical literacy (such as motor skills) may take more time to then impact on PA [61]. Further, if physical literacy is about developing capacity for PA over the life course, then it is important to consider follow up tests to ascertain a successful intervention. Also, there are many factors which affect PA, and therefore improving these other factors (i.e., the non-physical domains of physical literacy as well as environmental and other factors) may also feasibly have more of an effect on children’s PA [62].

Interestingly, articles included for review were designed to test these intervention outcomes (PA and physical components of physical literacy) as separate outcomes rather than to investigate whether an improvement in a physical literacy element is instrumental to an improvement in PA. This conundrum can also be found in the motor development literature. Nearly all motor skill interventions designed to improve motor skill development *and* PA, test for the outcomes separately rather than assessing whether the improvement in motor skills relates to any improvement in PA (or the reverse) [63]. This means that even if we do see an improvement in both outcomes, we cannot attribute one change to the change in the other variable. This is complicated by whether physical literacy is situated as *resulting in* PA or whether PA *is conceptualised as part of* or *preceding* physical literacy [64]. The divergence between studies included in the current review in how PA is treated reflect these different conceptual approaches. We recommend that future analyses match the conceptual framework of the researcher in terms of the treatment of the constructs of PA and physical literacy.

### Future Research Directions

Researchers and practitioners should consider creating holistic interventions which aim to improve and assess all facets of physical literacy, especially those elements less examined in the affective, cognitive, and social domains [15]. As tools to assess physical literacy (and physical activity) keep developing and amassing more validity and reliability evidence across different populations, this will help in being able to determine the success of programs [58]. In the future, it will be important to be able to tease out the intervention characteristics which lead to efficacy such as effective dose (length, frequency, duration). This is particularly important given the complexity of the school system, the multiple settings within schools, and the many interacting levels of influence in these settings. Future studies should therefore also focus on collecting data on implementation (such as acceptability, feasibility, fidelity) as this will be important to understand child and school outcomes, as well as participation pathways in sport and PA [65]. All included studies were conducted with typically developing children, indicating a knowledge gap with respect to school-based physical literacy interventions for children living with disability or a medical condition. Likewise, most studies were conducted with primary/elementary school-aged children, with only two targeting children in the junior high/ middle school age groups, suggesting physical literacy interventions are needed for children in older age groups. While outside the scope of this review, an important consideration of any physical literacy intervention in the school setting would be the engagement and upskilling of schools and educators around what is physical literacy and the importance of physical literacy within the school/sport setting [58]. This will assist with sustainability outcomes and effectiveness of any intervention.

### Strengths and Limitations

The strength of the current review is the rigorous definition and application of included studies. We specifically defined what we meant by physical literacy, and rigorously followed and mapped our inclusion criteria to an associated framework. It was not possible to perform any meta-analyses due to the diversity of outcomes and the limited number of studies and analyses investigating particular outcomes. We did, however, collate results according to all the tests performed rather than simply focusing on results in the expected direction which can lead to a bias in the literature of positive effect [66].

## Conclusions

Our findings provide support for holistic physical literacy interventions improving the physical components of children’s physical literacy in school settings. In the included quantitative studies, we found mixed evidence for holistic physical literacy interventions improving total physical literacy and the affective and cognitive domains/elements of physical literacy. We found no evidence for holistic physical literacy interventions improving the social components of children’s physical literacy (although this was understudied and not included in most physical literacy definitions or assessment tools) or other outcomes such as cognitive performance or PA (which most studies did assess), although qualitative findings reported benefits across all domains of the APLF and for PA. These findings can be used in the development, delivery, and evaluation of future physical literacy interventions.

## Electronic Supplementary Material

Below is the link to the electronic supplementary material.

Supplementary Material 1

## Data Availability

All data generated or analysed during this study are included in this published article (and its supplementary information files).

## Abbreviations

APLF: Australian Physical Literacy Framework
CAPL: Canadian Assessment of Physical Literacy
CASP: Critical appraisal skills programme
ESM: Electronic supplementary material
MFT: Multi-stage fitness test
PA: Physical activity
PACES: Physical activity enjoyment scale
PE: Physical education
PFL: Passport for life
PLAY: Physical literacy assessment for youth
PRISMA: Preferred Reporting Items for Systematic Reviews and Meta-Analyses
PROSPERO: International prospective register of systematic reviews
TMGD-2: Test of gross motor development 2
