# Supplementary material for: Effects of Holistically Conceptualised School-Based Interventions on Children’s Physical Literacy, Physical Activity, and Other Outcomes: A Systematic Review
Source: Sports Med Open. 2024 Sep 27;10:105. doi: 10.1186/s40798-024-00766-w (PMC11436493; doi:10.1186/s40798-024-00766-w)
Supplement: Supplementary file 1 — Supplementary Material 1 [file 40798_2024_766_MOESM1_ESM.docx]

# Table S1: PRISMA 2020 Statement

| **Section and Topic** | **Item #** | **Checklist item** | **Location where item is reported** |
| --- | --- | --- | --- |
| **TITLE** | | |  |
| Title | 1 | Identify the report as a systematic review. | Page 1 |
| **ABSTRACT** | | |  |
| Abstract | 2 | See the PRISMA 2020 for Abstracts checklist. | Page 2 |
| **INTRODUCTION** | | |  |
| Rationale | 3 | Describe the rationale for the review in the context of existing knowledge. | Page 4-5 |
| Objectives | 4 | Provide an explicit statement of the objective(s) or question(s) the review addresses. | Page 5 |
| **METHODS** | | |  |
| Eligibility criteria | 5 | Specify the inclusion and exclusion criteria for the review and how studies were grouped for the syntheses. | Page 8-9, Table 1 |
| Information sources | 6 | Specify all databases, registers, websites, organisations, reference lists and other sources searched or consulted to identify studies. Specify the date when each source was last searched or consulted. | Page 6-7 |
| Search strategy | 7 | Present the full search strategies for all databases, registers and websites, including any filters and limits used. | ESM Table S2 |
| Selection process | 8 | Specify the methods used to decide whether a study met the inclusion criteria of the review, including how many reviewers screened each record and each report retrieved, whether they worked independently, and if applicable, details of automation tools used in the process. | Page 7-8 |
| Data collection process | 9 | Specify the methods used to collect data from reports, including how many reviewers collected data from each report, whether they worked independently, any processes for obtaining or confirming data from study investigators, and if applicable, details of automation tools used in the process. | Page 9-11 |
| Data items | 10a | List and define all outcomes for which data were sought. Specify whether all results that were compatible with each outcome domain in each study were sought (e.g. for all measures, time points, analyses), and if not, the methods used to decide which results to collect. | Page 11 |
|  | 10b | List and define all other variables for which data were sought (e.g. participant and intervention characteristics, funding sources). Describe any assumptions made about any missing or unclear information. | Page 9-11 |
| Study risk of bias assessment | 11 | Specify the methods used to assess risk of bias in the included studies, including details of the tool(s) used, how many reviewers assessed each study and whether they worked independently, and if applicable, details of automation tools used in the process. | Page 10 |
| Effect measures | 12 | Specify for each outcome the effect measure(s) (e.g. risk ratio, mean difference) used in the synthesis or presentation of results. | Page 11 |
| Synthesis methods | 13a | Describe the processes used to decide which studies were eligible for each synthesis (e.g. tabulating the study intervention characteristics and comparing against the planned groups for each synthesis (item #5)). | Page 11-12 |
|  | 13b | Describe any methods required to prepare the data for presentation or synthesis, such as handling of missing summary statistics, or data conversions. | Page 12-13 |
|  | 13c | Describe any methods used to tabulate or visually display results of individual studies and syntheses. | Page 12-13 |
|  | 13d | Describe any methods used to synthesize results and provide a rationale for the choice(s). If meta-analysis was performed, describe the model(s), method(s) to identify the presence & extent of statistical heterogeneity, & software package(s) used. | Page 12-13 |
|  | 13e | Describe any methods used to explore possible causes of heterogeneity among study results (e.g. subgroup analysis, meta-regression). | N/a |
|  | 13f | Describe any sensitivity analyses conducted to assess robustness of the synthesized results. | N/a |
| Reporting bias assessment | 14 | Describe any methods used to assess risk of bias due to missing results in a synthesis (arising from reporting biases). | N/a |
| Certainty assessment | 15 | Describe any methods used to assess certainty (or confidence) in the body of evidence for an outcome. | N/a |
| **RESULTS** | | |  |
| Study selection | 16a | Describe the results of the search and selection process, from the number of records identified in the search to the number of studies included in the review, ideally using a flow diagram. | Page 13, Figure 1 |
|  | 16b | Cite studies that might appear to meet the inclusion criteria, but which were excluded, and explain why they were excluded. | Page 13, Figure 1, ESM Table S4 |
| Study characteristics | 17 | Cite each included study and present its characteristics. | Page 14-16, Table 2, ESM Table S5 |
| Risk of bias in studies | 18 | Present assessments of risk of bias for each included study. | Page 16, ESM Table S6 |
| Results of individual studies | 19 | For all outcomes, present, for each study: (a) summary statistics for each group (where appropriate) and (b) an effect estimate and its precision (e.g. confidence/credible interval), ideally using structured tables or plots. | ESM Table S5 |
| Results of syntheses | 20a | For each synthesis, briefly summarise the characteristics and risk of bias among contributing studies. | Page 16-24, Table 3, 4, 5 |
|  | 20b | Present results of all statistical syntheses conducted. If meta-analysis was done, present for each the summary estimate and its precision (e.g. confidence/credible interval) and measures of statistical heterogeneity. If comparing groups, describe the direction of the effect. | Table 3, 4 |
|  | 20c | Present results of all investigations of possible causes of heterogeneity among study results. | N/a |
|  | 20d | Present results of all sensitivity analyses conducted to assess the robustness of the synthesized results. | N/a |
| Reporting biases | 21 | Present assessments of risk of bias due to missing results (arising from reporting biases) for each synthesis assessed. | N/a |
| Certainty of evidence | 22 | Present assessments of certainty (or confidence) in the body of evidence for each outcome assessed. | N/a |
| **DISCUSSION** | | |  |
| Discussion | 23a | Provide a general interpretation of the results in the context of other evidence. | Page 24-28 |
|  | 23b | Discuss any limitations of the evidence included in the review. | Page 30 |
|  | 23c | Discuss any limitations of the review processes used. | Page 30 |
|  | 23d | Discuss implications of the results for practice, policy, and future research. | Page 29-30 |
| **OTHER INFORMATION** | | |  |
| Registration and protocol | 24a | Provide registration information for the review, including register name and registration number, or state that the review was not registered. | Abstract  Page 6 |
|  | 24b | Indicate where the review protocol can be accessed, or state that a protocol was not prepared. | Page 6 |
|  | 24c | Describe and explain any amendments to information provided at registration or in the protocol. | N/a |
| Support | 25 | Describe sources of financial or non-financial support for the review, and the role of the funders or sponsors in the review. | Page 32 |
| Competing interests | 26 | Declare any competing interests of review authors. | Page 32 |
| Availability of data, code and other materials | 27 | Report which of the following are publicly available and where they can be found: template data collection forms; data extracted from included studies; data used for all analyses; analytic code; any other materials used in the review. | Tables 2-5, ESM Tables S5-S8 (template data collection forms; data extracted from included studies; data used for all analyses) |

*From:*  Page MJ, McKenzie JE, Bossuyt PM, Boutron I, Hoffmann TC, Mulrow CD, et al. The PRISMA 2020 statement: an updated guideline for reporting systematic reviews. BMJ 2021;372:n71. doi: 10.1136/bmj.n71

# Table S2: Database search strategy

A systematic search across seven scientific databases (APA PsycINFO, EMBASE, ERIC, CINAHL Complete, Global Health, MEDLINE Complete, SPORTDiscus with Full Text) was conducted to identify either studies or reviews (narrative or systematic) of school-based physical literacy interventions. The search strategy, which combined terms for ‘child, ‘school’, ‘physical literacy, and ‘intervention’, was developed and adapted for each database by a university health librarian with expertise in advanced database searching. The search was conducted on 25 July 2022 and restricted to peer-reviewed English language articles published in the previous five years (1 January 2017 to 25 July 2022). An updated database search was conducted on 14 November 2023.

**Final search strategy – APA PsycInfo via EBSCOhost**

| **#** | **Query** |
| --- | --- |
| S13 | S10 AND S11  Limiters – Publication Year: 2017-2022 |
| S12 | S10 AND S11 |
| S11 | TI “physical* litera*” OR AB “physical* litera*” |
| S10 | S1 OR S2 OR S3 OR S4 OR S5 OR S6 OR S7 OR S8 OR S9 |
| S9 | DE "Physical Education" |
| S8 | DE "Cooperating Teachers" OR DE "Elementary School Teachers" OR DE "High School Teachers" OR DE "Junior High School Teachers" OR DE "Middle School Teachers" OR DE "Preservice Teachers" OR DE "Resource Teachers" OR DE "Special Education Teachers" OR DE "Student Teachers" OR DE "Teachers" OR DE "Instructional Media" OR DE "Advance Organizers" OR DE "Educational Audiovisual Aids" OR DE "Reading Materials" OR DE "Teaching Machines" OR DE "Textbooks" OR DE "Cooperative Learning" OR DE "Bilingual Education" OR DE "Teacher Student Interaction" OR DE "Teaching" OR DE "Teaching Methods" OR DE "Advance Organizers" OR DE "Audiovisual Instruction" OR DE "Computer Assisted Instruction" OR DE "Directed Discussion Method" OR DE "Discovery Teaching Method" OR DE "Educational Field Trips" OR DE "Experiential Learning" OR DE "Group Instruction" OR DE "Individualized Instruction" OR DE "Lecture Method" OR DE "Lesson Plans" OR DE "Montessori Method" OR DE "Nondirected Discussion Method" OR DE "Open Classroom Method" OR DE "Problem Based Learning" OR DE "Programmed Instruction" OR DE "Scaffolding" OR DE "Service Learning" OR DE "Team Teaching Method" OR DE "Tutoring" |
| S7 | DE "Intermediate School Students" OR DE "Primary School Students" OR DE "Students" OR DE "Elementary School Students" OR DE "High School Students" OR DE "International Students" OR DE "Junior High School Students" OR DE "Middle School Students" OR DE "Special Education Students" OR DE "Transfer Students" |
| S6 | DE "Boarding Schools" OR DE "Charter Schools" OR DE "Elementary Schools" OR DE "High Schools" OR DE "Junior High Schools" OR DE "Middle Schools" OR DE "Nongraded Schools" OR DE "Campuses" OR DE "School Environment" OR DE "Schools" OR DE "School Facilities" OR DE "Classroom Environment" |
| S5 | TI “physical educat*” OR AB “physical educat*” |
| S4 | TI teach* OR AB teach* |
| S3 | TI student* OR AB student* |
| S2 | TI pupil* OR AB pupil* |
| S1 | TI school* OR AB school* |

**Final search strategy – CINAHL Complete via EBSCOhost**

| **#** | **Query** |
| --- | --- |
| S13 | S10 AND S11  Limiters - Date Published: 20170601-20220631 |
| S12 | S10 AND S11 |
| S11 | TI “physical* litera*” OR AB “physical* litera*” |
| S10 | S1 OR S2 OR S3 OR S4 OR S5 OR S6 OR S7 OR S8 OR S9 |
| S9 | (MH "Physical Education and Training+") |
| S8 | (MH "Teachers") OR (MH "Teaching") OR (MH "Teaching Methods+") OR (MH "Teaching Materials+") |
| S7 | (MH "Students") OR (MH "Students with Disabilities") OR (MH "Students, Foreign") OR (MH "Students, Middle School") OR (MH "Students, Minority") OR (MH "Students, Non-Traditional") OR (MH "Students, High School") |
| S6 | (MH "Schools") OR (MH "Schools, Elementary") OR (MH "Schools, Middle") OR (MH "Schools, Secondary") OR (MH "Schools, Special") |
| S5 | TI “physical educat*” OR AB “physical educat*” |
| S4 | TI teach* OR AB teach* |
| S3 | TI student* OR AB student* |
| S2 | TI pupil* OR AB pupil* |
| S1 | TI school* OR AB school* |

**Final search strategy – ERIC via EBSCOhost**

| **#** | **Query** |
| --- | --- |
| S13 | S10 AND S11  Limiters - Date Published: 20170601-20220631 |
| S12 | S10 AND S11 |
| S11 | TI “physical* litera*” OR AB “physical* litera*” |
| S10 | S1 OR S2 OR S3 OR S4 OR S5 OR S6 OR S7 OR S8 OR S9 |
| S9 | DE "Dance" OR DE "Aquatic Sports" OR DE "Extramural Athletics" OR DE "Intramural Athletics" OR DE "Racquet Sports" OR DE "Team Sports" OR DE "Track and Field" OR DE "Exercise" OR DE "Physical Education" OR DE "Adapted Physical Education" OR DE "Movement Education" OR DE "College Athletics" OR DE "Physical Activities" OR DE "Physical Education Facilities" OR DE "Physical Education Teachers" OR DE "Physical Recreation Programs" OR DE "Recreational Activities" OR DE "Womens Athletics" |
| S8 | DE "High School Teachers" OR DE "Junior High School Teachers" OR DE "Child Development Specialists" OR DE "Bilingual Teacher Aides" OR DE "Teaching Styles" OR DE "Teachers" OR DE "African American Teachers" OR DE "Beginning Teachers" OR DE "Bilingual Teachers" OR DE "Catholic Educators" OR DE "Cooperating Teachers" OR DE "Early Childhood Teachers" OR DE "Elementary School Teachers" OR DE "Experienced Teachers" OR DE "Lay Teachers" OR DE "Master Teachers" OR DE "Middle School Teachers" OR DE "Minority Group Teachers" OR DE "Physical Education Teachers" OR DE "Public School Teachers" OR DE "Remedial Teachers" OR DE "Resource Teachers" OR DE "Secondary School Teachers" OR DE "Special Education Teachers" OR DE "Student Teachers" OR DE "Substitute Teachers" OR DE "Teacher Interns" OR DE "Teacher Researchers" OR DE "Television Teachers" OR DE "White Teachers" OR DE "One Teacher Schools" OR DE "Preservice Teachers" OR DE "Specialists" OR DE "Teacher Aides" OR DE "Teacher Attitudes" OR DE "Teacher Behavior" OR DE "Teacher Characteristics" OR DE "Teacher Collaboration" OR DE "Teacher Competencies" OR DE "Teacher Developed Materials" OR DE "Teacher Effectiveness" OR DE "Teacher Empowerment" OR DE "Teacher Evaluation" OR DE "Teacher Influence" OR DE "Teacher Leadership" OR DE "Teacher Made Tests" OR DE "Teacher Participation" OR DE "Teacher Persistence" OR DE "Teacher Placement" OR DE "Teacher Role" OR DE "Teacher Student Relationship" |
| S7 | DE "Junior High School Students" OR DE "Advanced Students" OR DE "African American Students" OR DE "American Indian Students" OR DE "Asian American Students" OR DE "Bilingual Students" OR DE "Commuting Students" OR DE "Elementary School Students" OR DE "Foreign Students" OR DE "Full Time Students" OR DE "Hispanic American Students" OR DE "Low Income Students" OR DE "Middle School Students" OR DE "Minority Group Students" OR DE "Pregnant Students" OR DE "Self Supporting Students" OR DE "Special Needs Students" OR DE "Student Athletes" OR DE "Student Volunteers" OR DE "Terminal Students" OR DE "White Students" OR DE "Transfer Students" OR DE "Continuation Students" OR DE "Students" OR DE "Students with Disabilities" OR DE "High School Freshmen" OR DE "High School Students" OR DE "Secondary School Students" |
| S6 | DE "Schools" OR DE "Bilingual Schools" OR DE "Boarding Schools" OR DE "Colleges" OR DE "Community Schools" OR DE "Consolidated Schools" OR DE "Correspondence Schools" OR DE "Day Schools" OR DE "Disadvantaged Schools" OR DE "Elementary Schools" OR DE "Experimental Schools" OR DE "Folk Schools" OR DE "Free Schools" OR DE "International Schools" OR DE "Laboratory Schools" OR DE "Magnet Schools" OR DE "Middle Schools" OR DE "Military Schools" OR DE "Montessori Schools" OR DE "Multiunit Schools" OR DE "Neighborhood Schools" OR DE "Open Plan Schools" OR DE "Preschools" OR DE "Private Schools" OR DE "Professional Development Schools" OR DE "Public Schools" OR DE "Racially Balanced Schools" OR DE "Regional Schools" OR DE "Rural Schools" OR DE "Schools of Education" OR DE "Secondary Schools" OR DE "Single Sex Schools" OR DE "Slum Schools" OR DE "Small Schools" OR DE "Special Schools" OR DE "State Schools" OR DE "Suburban Schools" OR DE "Summer Schools" OR DE "Traditional Schools" OR DE "Urban Schools" OR DE "Virtual Schools" OR DE "Vocational Schools" OR DE "Year Round Schools" |
| S5 | TI “physical educat*” OR AB “physical educat*” |
| S4 | TI teach* OR AB teach* |
| S3 | TI student* OR AB student* |
| S2 | TI pupil* OR AB pupil* |
| S1 | TI school* OR AB school* |

**Final search strategy – Global Health via EBSCOhost**

| **#** | **Query** |
| --- | --- |
| S13 | S10 AND S11  Limiters – Publication Year: 20170601-20220631 |
| S12 | S10 AND S11 |
| S11 | TI “physical* litera*” OR AB “physical* litera*” |
| S10 | S1 OR S2 OR S3 OR S4 OR S5 OR S6 OR S7 OR S8 OR S9 |
| S9 | DE "physical education" |
| S8 | DE "student teacher relationships" OR DE "teachers" OR DE "teaching materials" OR DE "teaching methods" OR DE "educational games" OR DE "field trips" OR DE "individualized instruction" OR DE "multimedia instruction" OR DE "role playing" OR DE "team teaching" OR DE "teaching" |
| S7 | DE "students" OR DE "foreign students" OR DE "high school students" OR DE "junior high school students" |
| S6 | DE "elementary schools" OR DE "high schools" OR DE "private schools" OR DE "public schools" OR DE "schools" OR DE "elementary education" OR DE "primary education" OR DE "secondary education" OR DE "students" |
| S5 | TI “physical educat*” OR AB “physical educat*” |
| S4 | TI teach* OR AB teach* |
| S3 | TI student* OR AB student* |
| S2 | TI pupil* OR AB pupil* |
| S1 | TI school* OR AB school* |

**Final search strategy – MEDLINE Complete via EBSCOhost**

| **#** | **Query** |
| --- | --- |
| S13 | S10 AND S11  Limiters - Date of Publication: 20170601-20220631 |
| S12 | S10 AND S11 |
| S11 | TI “physical* litera*” OR AB “physical* litera*” |
| S10 | S1 OR S2 OR S3 OR S4 OR S5 OR S6 OR S7 OR S8 OR S9 |
| S9 | (MH "Physical Education and Training") |
| S8 | (MH "School Teachers") OR (MH "Teaching") OR (MH "Teaching Materials+") |
| S7 | (MH "Students") |
| S6 | (MH "Schools") |
| S5 | TI “physical educat*” OR AB “physical educat*” |
| S4 | TI teach* OR AB teach* |
| S3 | TI student* OR AB student* |
| S2 | TI pupil* OR AB pupil* |
| S1 | TI school* OR AB school* |

**Final search strategy – SPORTDiscus with Full Text via EBSCOhost**

| **#** | **Query** |
| --- | --- |
| S13 | S10 AND S11  Limiters – Published Date: 20170601-20220631 |
| S12 | S10 AND S11 |
| S11 | TI “physical* litera*” OR AB “physical* litera*” |
| S10 | S1 OR S2 OR S3 OR S4 OR S5 OR S6 OR S7 OR S8 OR S9 |
| S9 | DE "PHYSICAL education" OR DE "COACHING (Athletics)" OR DE "DRILLS (Practice)" OR DE "FIELD days (Education)" OR DE "FUNCTIONAL training" OR DE "MOTOR learning" OR DE "MOVEMENT education" OR DE "MUSIC in physical education" OR DE "PHYSICAL Education Attitude Inventory" OR DE "PHYSICAL Education Teacher Assessment Instrument" OR DE "PHYSICAL education (Elementary)" OR DE "PHYSICAL education (Middle school)" OR DE "PHYSICAL education (Primary)" OR DE "PHYSICAL education (Secondary)" OR DE "PHYSICAL education for children" OR DE "PHYSICAL education for girls" OR DE "PHYSICAL education for older people" OR DE "PHYSICAL education for people with disabilities" OR DE "PLAYGROUND games" OR DE "PRACTICE (Sports)" OR DE "SCHOOL sports" OR DE "SPORTS clinics" OR DE "SWIMMING for children -- Training" |
| S8 | DE "PHYSICAL education teachers" OR DE "TEACHING" OR DE "TEACHERS" |
| S7 | (DE "SCHOOLS") AND (DE "STUDENTS" OR DE "SCHOOL children" OR DE "STUDENTS with disabilities") |
| S6 | DE "SCHOOLS" |
| S5 | TI “physical educat*” OR AB “physical educat*” |
| S4 | TI teach* OR AB teach* |
| S3 | TI student* OR AB student* |
| S2 | TI pupil* OR AB pupil* |
| S1 | TI school* OR AB school* |

**Final search strategy – Embase via Embase.com**

| **#** | **Query** |
| --- | --- |
| #13 | #12 AND (2017:py OR 2018:py OR 2019:py OR 2020:py OR 2021:py OR 2022:py) |
| #12 | #10 AND #11 |
| #11 | 'physical* litera*':ab,ti |
| #10 | #1 OR #2 OR #3 OR #4 OR #5 OR #6 OR #7 OR #8 OR #9 |
| #9 | 'physical education'/exp |
| #8 | 'teacher'/de OR 'school teacher'/de OR 'teaching assistant'/de OR 'teaching'/de OR 'teaching'/de |
| #7 | 'student'/de OR 'athletic training student'/de OR 'disabled student'/de OR 'elementary student'/de OR 'foreign student'/de OR 'high school student'/de OR 'middle school student'/de OR 'student athlete'/de |
| #6 | ‘school’/de OR ‘high school’/de OR ‘middle school’/de OR ‘primary school’/de OR ‘primary education’/de OR ‘secondary education’/de |
| #5 | 'physical* educat*':ab,ti |
| #4 | teach*:ab,ti |
| #3 | student*:ab,ti |
| #2 | pupil*:ab,ti |
| #1 | school*:ab,ti |

# Table S3: Reviews of physical literacy interventions

| **Author(s)** | **Review type** | **Title** | **Additional studies meeting inclusion criteria (not already identified through database searches)** |
| --- | --- | --- | --- |
| Anico et al. (2022) | Systematic review | The effectiveness of school-based run/walk programmes to develop physical literacy and physical activity components in primary school children: A systematic review | None |
| Barbosa Filho et al. (2021) | Scoping review | Scoping Review on Interventions for Physical Activity and Physical Literacy Components in Brazilian School-Aged Children and Adolescents | None |
| Bopp et al. (2022) | Systematic review | Physical Literacy Research in the United States: A Systematic Review of Academic Literature | None |
| Carl et al. (2022a) | Systematic review | How are physical literacy interventions conceptualized? A systematic review on intervention design and content | Invernizzi et al. (2019); Bremer et al. (2020) |
| Carl et al. (2022b) | Systematic review and meta-analysis | The Effectiveness of Physical Literacy Interventions: A Systematic Review with Meta‑Analysis | Invernizzi et al. (2019); Bremer et al. (2020) |
| Jimenez-Garcia et al. (2023) | Systematic review and Meta-analysis | Effects of Multicomponent Injury Prevention Programs on Children and Adolescents' Fundamental Movement Skills: A Systematic Review With Meta-Analyses | None |
| Liu and Chen (2021) | Narrative review | Physical literacy in children and adolescents: Definitions, assessments, and interventions | None |
| Miyahara (2020) | Narrative Critical review | Physical Literacy as A Framework of Assessment and Intervention for Children and Youth with Developmental Coordination Disorder: A Narrative Critical Review of Conventional Practice and Proposal for Future Directions | None |
| Pushkarenko et al. (2021) | Scoping review | Physical literacy and inclusion: A scoping review of the physical literacy literature inclusive of individuals experiencing disability | None |
| Saxena and Shikako Thomas (2020) | Realist review | Physical literacy programs for children with disabilities: a realist review | None |

# Table S4: School-based physical literacy interventions excluded from review at second stage of full-text screening

| Exclusion reason | Study Author (year) | Location |
| --- | --- | --- |
| *Only 1(physical) domain of PL assessed* | Chapelski et al. (2023) | Canada |
|  | Johnstone et al. (2017) | Scotland, UK |
|  | Kozera (2017) | Canada |
|  | Lightner et al. (2023) | USA |
|  | Wright et al. (2020) | Canada |
|  | Zhang et al. (2023) | China |
| *Only 1 (cognitive) domain of PL assessed* | Deutsch et al. (2022) | USA |
| *Only 2 (physical and cognitive) domains of PL assessed* | Alagul et al. (2012) | Turkey |
|  | Santos et al. (2017) | Portugal |
|  | Strobl et al. (2020) | Germany |
| *Only 2 (physical and affective) domains of PL assessed* | Colella and Bonasia (2019) | Italy |
|  | Gavigan et al. (2021) | Ireland |
|  | Hulteen et al. (2023) | Canada |
|  | Lloyd (2016) | Canada |
|  | Pullen et al. (2020) | Wales, UK |
|  | Wainwright et al. (2018) | Wales, UK |
| *Insufficient information reported for results* | Wahyuni et al. (2023) | Indonesia |
| *Intervention not grounded in movement* | Everley (2021) | England, UK |

Abbreviations: PL = physical literacy; UK = United Kingdom; USA = United States of America

# Table S5: Description of included studies and summary of intervention outcomes

| **Authors year, location** | **Research design** | **Population (n, age, gender)** | **School setting** | **Intervention group** | **Control group** | **Intervention Evaluation (Quant. Vs. Qual.)** | **PL and other outcome assessment tool(s)** | **Summary of intervention outcomes**  **(and/or qualitative findings)** | |
| --- | --- | --- | --- | --- | --- | --- | --- | --- | --- |
|  |  |  |  |  |  |  |  | **Within group results** | **Between group results** |
| Bremer et al. (2020)  **Canada** | Cluster RCT  (pre-post; 12-week intervention) | n=90; 7-13 years old; 53% male. | Afterschool program | PL focused afterschool program (15mins FMS stations + 15mins active games)  (n=47; mean age 9.1 ± 1.4 years) | Usual afterschool program  (n=43; mean age 10.5 ± 1.8 years) | Quantitative (motor skills tests, questionnaire) | PLAY*fun*  PLAY*self*  IMI (motivation and enjoyment)  Self-efficacy  Other-efficacy: Peer, Leader RISE (relation-inferred self-efficacy): Peer, Leader |  | Significant intervention effects on enjoyment (0.95 [0.43], p<0.03), other-efficacy (leader) (2.92 [0.66], p>0.001) and RISE (leader) (1.14 [0.56], p<0.05), IV group reporting higher scores at post-intervention NS intervention effects on 6/9 outcomes: motor competence, PLAYfun average score (-3.99 (SE2.36], p<0.10), self-efficacy (0.07 [SE0.38] p<0.85), motivation (0.56 [0.38], p<0.14), PLAYself total score (0.61 [4.78], p<0.90), other efficacy (peer) (0.07 [0.81], p<0.93), RISE (peer) (1.36 [0.81], p<0.10). |
| Caldwell et al. (2022)  **Canada** | Mixed methods evaluation study (post-intervention: ~12 weeks/one semester) | n=14; mean age 9.25 years;  Grades 4–6; 45% male. | Afterschool program | Build Our Kids Success (BOKS) (BOKS 2023)  PA focused afterschool program (lesson plans, short movement bursts and movement-based games & activities)  (n=14)  Note: children eligible if attended ≥1 session/week | None | Qualitative (interviews)  Measured post-intervention.  Note: study design and data collection altered due to Covid – quantitative data did not meet eligibility criteria for this review. |  | Qualitative findings from participant interviews post-intervention indicated improvements across four PL domains, including muscular endurance, strength, engagement, enjoyment, knowledge, and PA participation. |  |
| Coyne et al. (2019)  **Canada** | Within subjects design (pre-post; 10-week intervention) | n=310; mean age 10.5 ± 1.0 years (range 8-12yrs); Grades 4-6; 50% male. | PE | PL based PE classes Fundamental movement skills program with a focus on track and field inspired games (The Run, Jump, Throw, Wheel [RJTW] Program) (n=310) | None | Quantitative (motor/fitness skill tests, questionnaire, heart rate monitoring) | CAPL-1  Heart rate (to assess physical activity as daily behaviour and % of PE class in light, moderate and vigorous intensity PA) |  | Significant intervention effects on PL (CAPL score +3.3 (±8.8) points, p<0.001, Cohens *d* 0.303; overall physical competence +1.9, p<0.001, Cohens *d* 0.914; knowledge and understanding +0.8, p<0.001, Cohen’s *d* 0.026), and PA (% PE in moderate PA) -2.33, p<0.016, Cohen’s *d* 0.285).  NS intervention effects on motivation and confidence, daily PA, % PE class in light, or vigorous activity |
| Farias et al. (2020)  **Portugal** | Participatory case study with retrospective evaluation  (one school year) | N=25; 12–14 years old; Grade 7; 64% male. | PE | Year-long sport education curriculum (Sport Education) with action research element (n=26) | None | Qualitative (Memory retrieval tour, Interviews, Survey, Focus group)  3-years post-intervention |  | Qualitative findings indicated improvements across four PL domains, including student’s enthusiasm and enjoyment of PE and PA. Increased physical competency, cognitive and relational elements were also reported. |  |
| Invernizzi et al. (2019)  **Italy** | Cluster RCT  (pre-post; 12-week intervention) | N=121; mean age 10.5 ± 0.5 years; Grade 5; 47% male. | PE | PE program based on multi-teaching approaches (n = 62) | Usual PE lessons (n=59) | Quantitative and qualitative (motor/fitness skill tests, questionnaires)  Qualitative (interviews with children, post only) | MFT  TGMD-2  PACES  SDQ  PAQ-C | Qualitative findings indicated self-reported improvements across two PL domains, including enjoyment of PA, and cognitive factors such as rules and reasoning. | Significant intervention effects on fitness (mean diff 1.383, [SE: 0.443], p<0.002, η^2^ 0.09), gross motor development (mean diff 5.441 [SE1.191], p<0.001, η^2^ 0.17), enjoyment (mean diff 1.90 [SE 0.080], p<0.020, η^2^ 0.96), and total physical activity (mean diff 0.345 [SE 0.109], p<0.002, η^2^ 0.09).  No improvement in physical self-perception (mean diff 0.039 [SE 0.068], p<0.568, η^2^ 0.00). |
| Kriellaars et al. (2019)  **Canada** | Prospective, clustered, quasi-experimental CT  (pre-post; ~12 weeks/one semester intervention) | N= 211; mean age 10.1 ± 0.8 years; Grade 4 and 5; 45% male. | PE | Circus arts instruction in PE class (n=110) | Usual PE instruction with traditional curriculum focusing on sports and small/medium sized games. (n = 101) | Quantitative (motor/fitness skill tests, questionnaire) | PLAY*fun*  PLAY*self*  PLAY*inventory* | Significant improvements in motor competence for both groups, with larger improvements seen in the intervention group. | Significant intervention effects on motor competence (*d*=0.512, P<0.01). Significant differences for Gr5 children in 15/18 tasks and for G4 children in 7/18 tasks.  Significant intervention effects on total number of physical activity pursuits (endpoint #: 26 vs 20, p<0.05)  Significant intervention effects on PL self-assessment score (P<0.05) |
| Li et al. (2022)  **Hong Kong** | 3-arm RCT *post intervention + 3-month follow-up. Between group (group x time) and within group (time) analysis  (13-week intervention) | N= 79; mean age 9.6 ± 0.61 years; 41% male. | Classroom sit-stand desks, active recess play | (1) A blended PL group-combined sit–stand desks and play-based recess (SSPlay; n=24)  (2) A single play-based group (Play; n=27) | Usual class schedules and lesson delivery (n=28) | Quantitative (motor/fitness skill tests, PA measurement, questionnaires, computer-based cognitive tests) at post intervention and 3-month follow-up. | CAPL-2: Chinese  (PACER, CAMSA, Pedometer, Questionnaire)  Accelerometer (GT3X+)  Wisconsin Card Sorting Test (WCST)  Tower of London Task (TLT) | Post-intervention (mean change [95%CI]):  SSPlay group: significant change in physical literacy components of competence (2.96 [5.21, 0.67]) knowledge and understanding (2.35 [3.57, 1.12]); cognitive flexibility components of total errors (24.0 [16.08, 31.92]) and perseverative errors (2.09 [0.75, 3.42]).  Play group: significant change in physical literacy components of competence (5.15 [6.85, 3.45]) knowledge and understanding (2.00 [3.17, 0.83]), daily behaviour (3.0 [4.17, 4.17, 1.29]); cognitive flexibility component of total errors (12.0 [4.19, 21.65]).  Follow-up (mean change [95%CI]):  SSPlay group: significant change in physical literacy components of competence (2.67 [4.81, 0.53]) knowledge and understanding (1.87 [3.10, 0.64]); cognitive flexibility components of total errors (22.78 [13.36, 32.21]) and perseverative errors (2.26 [0.82, 3.70]).  Play group: significant change in physical literacy components of competence (2.67 [4.97, 0.37]) knowledge and understanding (1.32 [2.28, 0.36]), cognitive flexibility component of total errors (21.88 [14.15, 29.61]), planning component of total correct (9.12 (16.89, 1.35). | Post intervention:  Significant intervention effects (group x time) on physical competence (p<0.02) and daily behaviour (p<0.001). No intervention effects on physical activity, motivation and confidence, knowledge and understanding cognitive flexibility and cognitive planning  Follow-up:  No intervention (group x time) effects on any outcomes |
| Liu and Chen (2022)  **USA** | Within subjects design  (pre-post; 8-week intervention) | n=49 (subsample from larger sample); aged 11 to 13 years; grade 6 – 8; 47% male. | PE | Pedagogical Workshops each with motivational and informational modules based on the Heart PL model (Learning in high-low performing dyads as determined by the CAPL-2), 4 sessions over 8 weeks (n=49) with low ‘beginning’-performing PL (n=26) and high ‘Excelling’-performing PL (n=23) | None | Quantitative (motor/fitness skill tests, PA measurement, questionnaires) Qualitative (focus groups with children pre & post) | CAPL-2  (PACER, CAMSA, Pedometer, Questionnaire) | The low PL group showed improvements in total PL and all PL domains. High-PL group improved in the cognitive domain only. Interview data indicated improved enjoyment and motivation for PA.  Low PL group: medium size improvements for total PL (mean pre-post difference: 7.42, Hedges g = 0.69), cognitive domain (mean difference; 0.83, Hedges g = 0.46, Physical domain (mean difference 1.71, Hedges g = 0.36), behavioural domain (mean difference: 1.61, Hedges g=0.35) and Affective domain (Hedges g = 0.34).  High PL group: small increase in cognitive domain (mean diff; 0.23, Hedges g=0.15); medium size declines in total PL (mean diff; -3.26, Hedges g = -0.46, behavioural domain (mean diff; -2.79, Hedges g = -0.53); small declines in physical domain (mean diff; -0.68, Hedges g = -0.19) and affective domain (mean diff; -0.04, Hedges g=-0.02).  Qualitative findings indicated self-reported improvements across four PL domains, including movement skills, strength, confidence, and motivation for PA | Significant group x time intervention effect for the behavioural domain (p<0.01, ηp2 =0.58) |
| Mandigo et al. (2019)  **Canada** | Within subjects design  (pre-post; 8-week intervention) | N= 22; Grades 5 to 8; 27% male. | Afterschool program | 8-week PlaySport program based on a Teaching Games for Understanding (TgfU) approach (n = 22) | None | Quantitative (motor/fitness skill tests, questionnaires) | PFL | Significant improvements in balance/stability (<0.001), cardiovascular endurance/fitness (p<0.001), diverse environments (p<0.003) and diverse interests (p<0.002) and feeling (p<0.050). No change in throwing, running, kicking, muscle endurance, thinking, interacting. |  |
| Mendoza-Muñoz et al. (2022)  **Spain** | Quasi-experimental pilot study  (pre-post; 4-week intervention) | N= 57; mean age 10.28 ± 0.43 years; 49% male. | Structured recess | 4-week AB recess program (experimental group [EG], n=28) | Usual recess (control group [CG], n=29) | Quantitative (motor/fitness skill tests, PA measurement, Questionnaires) | CAPL-2  (PACER, CAMSA, Pedometer, Questionnaire) | EG showed pre-post improvements (all p<0.001 except where stated) in overall PL points, self-reported PA (mean change [SD]; 7.11 [-1.11], p<0.044), Physical competence domain (including CAMSA and PACER points) (3.98 [-0.26]), Motivation and confidence domain (including predilection, adequacy, (p<0.023) intrinsic motivation and competence points) (1.14 [-0.48]), knowledge and understating domain (0.72 [-0.11]). No change in Daily behaviour domain, steps, or plant points.  CG showed significant pre-post declines (all p<0.001 except where stated) in Daily behaviour domain (including diary steps p<0.008), (-1.21 [-0.73]), Motivation and confidence domain (including adequacy, (p<0.037) intrinsic motivation and competence points) (-0.18 [-0.17]). No change in overall physical literacy, physical competence domain or knowledge and understanding domain. | Post intervention between group showed EG had greater (all p<0.001 except where stated) overall PL, physical competence domain (including CAMSA and plank points), Motivation and confidence domain (including predilection, adequacy, intrinsic motivation, and competence), Knowledge and understanding domain, and self-reported PA. No difference in daily behaviour domain, diary steps or PACER |
| Stoddart et al. (2021)  **Canada** | Quasi-experimental CT  (pre-post; 8-9-week intervention) | N= 131; mean age 10.28 ± 0.43 years; 50% male. | PE | 8–9-week PlitPE Intervention (n=81) | Usual practice in school (n=48) | Quantitative (motor/fitness skill tests, questionnaires) | PLAY*fun*,  PLAY*self*  PLAY*inventory* | IG pre-post:  Increase in average motor competence (d=0.88, p<0.001) and movement vocabulary (p<0.01). Significant increase in 5/7 PLAYfun measures,  CG pre-post:  Increase in average motor competence (d=0.39, p<0.01). Significant increase in 3/7 PLAYfun measures, | Significant difference between groups post-intervention for average motor competence (p<0.001, d=1.04) and environmental participation.  No differences (post) in PL self-description, PL score or self-reported PA (number of activities) for either group. |
| Telford et al. (2021)  **Australia** | Cluster RCT  (pre-post; 33-week intervention) | N= 318; mean age 10.4 ± 0.4 years; 49% male. | PE, active recess, active lessons | PEPL coach to support classroom teacher to teach PE and provide PL development (n=152) | Usual practice in school (n=166) | Quantitative (motor skill tests, PA measurement, questionnaires) &  Qualitative (Focus groups with children and teachers, post only) | TGMD-2  CY-PSPP  S-PACES  Accelerometer | Qualitative findings indicated self- and teacher-reported improvements across four PL domains, including increased confidence and motivation for PA. | Significant intervention effects for object control skills (p<0.008).Significant reduction in self-report sport competence (p<0.013) .  No effect on accelerometer counts or PA levels, self-perception of physical condition, physical self-worth, or PA enjoyment (all p>0.05) |

Table reports study characteristics and a summary of main outcomes. Within group results are reported by timepoint e.g. post IV (#weeks), follow-up (#weeks). Between group results are reported by timepoint e.g. post IV (#weeks), follow-up (#weeks). Qualitative findings are reported in the ‘within group’ column. Abbreviations: AB = Active break; CAMSA = Canadian Agility and Movement Skill Assessment; CAPL-2 = Canadian Assessment of Physical Literacy (version 2); CAPL-2: Chinese = Canadian Assessment of Physical Literacy (Chinese version); CG = Control group; CT = Controlled trial; CY-PSPP = Children and Youth Physical Self-Perception Profile; Diff = Difference; EG = Experimental group; FMS = Fundamental movement skills; IG = Intervention group; IV = Intervention; MFT = Multistage Fitness Test; PA = physical activity; PACER = Progressive Aerobic Cardiovascular Endurance Run; PACES = Physical Activity Enjoyment Scale; PAQ-C = Physical activity questionnaire-children; PE = physical education; PEPL = Physical education and physical literacy approach; PFL = Passport For Life; PL = physical literacy; PLAY*coach* = Physical Literacy Assessment for Youth-teacher-report questionnaire; PLAY*fun* = Physical Literacy Assessment for Youth – motor competence test of 18 skills; PLAY*inventory* = Physical Literacy Assessment for Youth-behavioural self-report questionnaire; PLAY*parent* = Physical Literacy Assessment for Youth-parent-report questionnaire; PLAY*self* = Physical Literacy Assessment for Youth-self-evaluation questionnaire; PlitPE = physical literacy enriched physical education; Qual. = qualitative; Quant. = quantitative; RCT = Randomised control trial; RISE = relation-inferred self-efficacy; RJTW Program = run, jump, throw, wheel program; S-PACES = Shortened-Physical Activity Enjoyment Scale; SD = Standard Deviation; SDQ = Self-Description Questionnaire; SE = Standard error; SSPlay = Sit-Stand Play; TgfU = teaching games for understanding; TGMD-2 = Test of Gross Motor Development (version 2); TLT = Tower of London Task; WCST = Wisconsin Card Sorting Test.

# Table S6: Risk of bias assessment

Quantitative designs

| **Lead Author** | **Year** | **Journal** | **Baseline characteristics comparable** | **Randomisation clearly described and carried out** | **Validated measures of PL used** | **Dropout described** | **Dropout ≤20% for <6-m follow-up; ≤30% for ≥6-m follow-up** | **Outcome assessors blinded** | **Sample size/power calculation reported** | **Study adequately powered** | **Intention to treat analysis used** | **Potential confounders accounted** | **Participants followed for ≥6-m** | Positive | Negative | Not or insufficiently described | Not applic-able |
| --- | --- | --- | --- | --- | --- | --- | --- | --- | --- | --- | --- | --- | --- | --- | --- | --- | --- |
| Bremer | 2020 | IJERPH | Positive | Not or insufficiently described | Positive | Positive | Positive | Positive | Positive | Positive | Negative | Positive | Negative | 8 | 2 | 1 | 0 |
| Coyne | 2019 | APNM | Not applicable | Not applicable | Positive | Not applicable | Not applicable | Not or insufficiently described | Negative | Not or insufficiently described | Not applicable | Positive | Negative | 2 | 2 | 2 | 5 |
| Invernizzi | 2019 | Sustain-ability | Positive | Not or insufficiently described | Positive | Not or insufficiently described | Not or insufficiently described | Not or insufficiently described | Negative | Not or insufficiently described | Negative | Positive | Negative | 3 | 3 | 5 | 0 |
| Kriellaars | 2019 | JTPE | Positive | Not applicable | Positive | Positive | Positive | Positive | Positive | Positive | Negative | Positive | Negative | 8 | 2 | 0 | 1 |
| Li | 2022 | SM - O | Positive | Positive | Positive | Positive | Positive | Not or insufficiently described | Positive | Positive | Positive | Positive | Negative | 9 | 1 | 1 | 0 |
| Liu | 2022 | FiSAL | Not applicable | Not applicable | Positive | Positive | Negative | Not or insufficiently described | Negative | Not or insufficiently described | Negative | Positive | Negative | 3 | 4 | 2 | 2 |
| Mandigo | 2019 | JTPE | Not applicable | Not applicable | Positive | Positive | Positive | Not or insufficiently described | Negative | Not or insufficiently described | Negative | Negative | Negative | 3 | 4 | 2 | 2 |
| Mendoza-Muñoz | 2022 | IJERPH | Positive | Not applicable | Positive | Negative | Positive | Negative | Negative | Not or insufficiently described | Positive | Negative | Negative | 4 | 5 | 1 | 1 |
| Stoddart | 2021 | PESP | Not or insufficiently described | Not applicable | Positive | Positive | Positive | Positive | Negative | Negative | Negative | Negative | Negative | 4 | 5 | 1 | 1 |
| Telford | 2021 | PESP | Negative | Negative | Positive | Positive | Positive | Negative | Negative | Not or insufficiently described | Positive | Positive | Negative | 5 | 5 | 1 | 0 |

APNM = Applied Physiology and Nutritional Metabolism; FISAL = Frontiers in Sports and Active Living; JTPE = Journal of Teaching in Physical Education; IJERPH = International Journal of Environmental Research and Public Health; PESP = Physical Education and Sport Pedagogy; RQES = Research Quarterly for Exercise and Science; SM – O = Sports Medicine – Open.

Qualitative designs

| **Author** | **Year** | **Journal** | **Clear statement of aims** | **Qualitative methodology appropriate** | **Design appropriate to the aims** | **Clear and consistent theoretical underpinnings** | **Appropriate recruitment strategy** | **Data collected in a way that addressed the research** | **Relationship between researcher and participants** | **Ethical issues considered** | **Data analysis sufficiently rigorous** | **Clear statement of findings** | **Yes** | **No** | **Somewhat** | **Cannot tell** |
| --- | --- | --- | --- | --- | --- | --- | --- | --- | --- | --- | --- | --- | --- | --- | --- | --- |
| Caldwell | 2022 | IJERPH | Somewhat | Somewhat | Somewhat | Somewhat | Cannot tell | Yes | No | Yes | Somewhat | Somewhat | 2 | 1 | 6 | 1 |
| Farias | 2020 | RQES | Yes | Yes | Yes | Yes | Yes | Yes | No | Yes | Yes | Yes | 9 | 1 | 0 | 0 |
| Invernizzi | 2019 | Sustainability | Somewhat | Somewhat | No | No | Cannot tell | No | No | Yes | Somewhat | Yes | 2 | 4 | 3 | 1 |
| Liu | 2022 | FiSAL | Yes | Yes | Somewhat | Somewhat | Yes | Somewhat | No | Yes | Somewhat | Somewhat | 4 | 1 | 5 | 0 |
| Telford | 2021 | PESP | Yes | Yes | Yes | No | Yes | Somewhat | No | Yes | Somewhat | Somewhat | 5 | 2 | 3 | 0 |

FISAL = Frontiers in Sports and Active Living; IJERPH = International Journal of Environmental Research and Public Health; PESP = Physical Education and Sport Pedagogy; RQES = Research Quarterly for Exercise and Science.

# Table S7: Assessment instruments for physical literacy and other outcomes (quantitative designs)

|  | Bremer et al. (2020) | | Coyne et al. (2019) | Invernizzi et al. (2019) | | Kriellaars et al. (2019) | | Li et al. (2022) | | Liu and Chen (2022) | | Mandigo et al. (2019) | | Mendoza-Muñoz et al. (2022) | | Stoddart et al. (2021) | Telford et al. (2021) | |
| --- | --- | --- | --- | --- | --- | --- | --- | --- | --- | --- | --- | --- | --- | --- | --- | --- | --- | --- |
| **TOTAL PL**  (self-report & composite score) | PLAY*self* (CS4L (Canadian Sport for Life) 2014b)  (Self-report) | | CAPL-1 (Longmuir et al. 2015)  (Composite score) |  | | PLAY*self* (Canadian Sport for Life) 2014b)  (Self-report) | | CAPl-2 Chinese (Li et al. 2020)  (Composite score) | | CAPL-2 (Healthy Active Living and Obesity Research Group (HALO) 2017)  (Composite score) | |  | | CAPL-2 (Longmuir et al. 2018)  (Composite score) | | PLAY*self* (CS4L (Canadian Sport for Life) 2014b)  (Self-report) |  | |
| **Physical Domain** (objective measures & self-report) | PLAY*fun* (Canadian Sport for Life 2013)  (Combined motor competence)  PLAY*self* | | CAPL-1 (PACER, CAMSA, plank) | TGMD-2 (Ulrich 2004)  MFT (Winsley 2003) | | PLAY*fun*  (CS4L (Canadian Sport for Life) 2014a)  (Combined motor competence)  PLAY*self* | | CAPl-2 Chinese (PACER, CAMSA, plank) | | CAPL-2 (PACER, CAMSA, plank) | | PFL (Physical and Health Education Canada 2013) | | CAPL-2 (PACER, CAMSA, plank) | | PLAY*fun* (CS4L (Canadian Sport for Life) 2014a)  PLAY*self* | TGMD-2 (Ulrich 2004) | |
| **Psychological Domain**  (self-report, peer or leader-report) | PLAY*self*  Motivation and enjoyment (IMI) (Ryan 1982); Self-/ other-efficacy; RISE | | CAPL-1  (Self-report 4-items) | PACES (Carraro et al. 2008)  SDQ (Marsh and Shavelson 1985) | | PLAY*self* | | CAPl-2 Chinese (Self-report 4-items) | | CAPL-2  (Self-report 4-items) | | PFL | | CAPL-2  (Self-report 4-items) | | PLAY*self* | CY-PSPP (Welk et al. 1995),  S-PACES (Dishman et al. 2005) | |
| **Cognitive Domain**  (self-report) | PLAY*self* | | CAPL-1  (Self-report 5-items) |  | | PLAY*self* | | CAPl-2 Chinese (Self-report 5-items) | | CAPL-2  (Self-report 5-items) | | PFL | | CAPL-2  (Self-report 5-items) | | PLAY*self* |  | |
| **Social Domain** (self-report) |  | |  |  | |  | |  | |  | | PFL | |  | |  |  | |
| **OTHER OUTCOMES** | |  | | |  | |  | |  | |  | |  | |  | | |  |
| **Physical activity (Behavioural Domain)**  (self-report & device-based measures) |  | | CAPL-1  (Pedometer; PA Self-report 1-item) |  | | PLAY*inventory* | | CAPL-2 Chinese  (Pedometer; PA Self-report 1-item) | | CAPL-2  (Pedometer; PA Self-report 1-item) | | PFL | | CAPL-2  (Pedometer; PA Self-report 1-item) | | PLAY*inventory* |  | |
| **Physical activity** (self-report & device-based measures) |  | | HR monitors | PAQ-C (Crocker et al. 1997) | |  | | Accelerometer (GT3X+) | |  | |  | |  | |  | Accelerometer (wGT3X-BT) | |
| **Cognitive performance** (computer-based) |  | |  |  | |  | | WCST (Heaton et al. 1993)  TLT (Anderson et al. 1996) | |  | |  | |  | |  |  | |

Instruments for included studies are classified against the APLF in terms of which domains/elements they assess, a behavioural domain of physical literacy that includes physical activity, or wider area of interest e.g., cognitive performance. Abbreviations: CAMSA = Canadian agility and movement skills assessment; CAPL-1 / CAPL-2/ CAPL-2: Chinese = Canadian Assessment of Physical Literacy (version1/ version 2/ Chinese version); CY-PSPP = Children and Youth Physical Self-Perception Profile; HR = heart rate; IMI = Intrinsic Motivation Inventory; MFT = Multistage Fitness Test; PA = physical activity; PACER = Progressive Aerobic Cardiovascular Endurance Run; PACES = Physical Activity Enjoyment Scale; PAQ-C = Physical Activity Questionnaire-Children; PFL = Passport For Life; PL = physical literacy; PLAY*fun* = Physical Literacy Assessment for Youth - motor competence test of 18 skills; PLAY*inventory* = Physical Literacy Assessment for Youth-behavioural self-report questionnaire; PLAY*self* = Physical Literacy Assessment for Youth-self-evaluation questionnaire; Qn = question; RISE = Relation-Inferred Self-Efficacy; S-PACES = Shortened-Physical Activity Enjoyment Scale; SDQ = Self-Description Questionnaire; TGMD-2 = Test of Gross Motor Development (version 2); TLT = Tower of London Task; WCST = Wisconsin Card Sorting Test.

# Table S8: Qualitative data collection methodology

| ***Authors*** | **Caldwell et al. (2022)** | **Farias et al. (2020)** | **Invernizzi et al. (2019)** | **Liu and Chen (2022)** | **Telford et al. (2021)** |
| --- | --- | --- | --- | --- | --- |
| ***Study design*** | Mixed methods | Qualitative multi-method | Mixed methods | Mixed methods | Mixed methods |
| ***Qualitative data collection method(s)*** | Interviews (online)  Post-intervention | Memory retrieval techniques, retrospective survey, semi-structured individual, and focus groups interviews  3 years post-intervention | Semi-structured interviews  Post-intervention | Focus groups  Pre- and post-intervention | Focus groups  Post-intervention |
| ***Participants*** | Children, parents, program leaders (IG only) | Children, Class Director (IG only) | Children (IG and CG) | Children (IG only) | Children, Teachers (IG only) |
| ***Qualitative research aims/ questions*** | To examine the impact of the BOKS (after-school) program from the various participant’s perspectives (child, parent, program leader) e.g., perceptions of children’s enjoyment, participation, outcomes, and barriers | To examine the impact of student participation in a year-long Sport Education curricular experience on physical literacy development, particularly students’ reported motivation, attitudes, and disposition to participate in PE and sport, and their re-actualizations of a healthy sport culture following the Sport Education experience. | To further analyse the effect of the MTA and S-PE approaches on the results. Questions related to perceived level of satisfaction about approaches and the primary school classroom teacher or PE students who conducted the lessons. | To capture PL journeys in light of participation in an SDT-guided pedagogical workshop. Specifically, questions were asked about their participation, experiences, perceptions, and motivation of physical activities; as well as the changes over time. | To explore social domain impacts and gather opinions on the experiences, impacts and implementation of the PEPL approach, and its effect on total school appreciation of PE and PA. |
| ***Data analysis*** | Inductive thematic analysis | Thematic analysis | Inductive coding, categorisation, and visualisation | Inductive thematic analysis  Deductive content analysis | Inductive content analysis |
| ***Trustworthiness/ Rigour*** | Investigator triangulation, Collaborative review of themes | Data triangulation, crosschecking, collaborative interpretational analysis | Not specified | Method triangulation through interviews, observations, and written records | Inter-rater checking of the coding, consensus validation |

Table summarises the five included studies that employed qualitative data collection methods to assess children’s physical literacy development. CG – control group; IG – intervention group; MTA = multi-teaching approaches; PA – physical activity; PE = physical education; PL = physical literacy; PEPL = physical education physical literacy; S-PE = standard PE.

# References

Alagul O, Gursel F and Keske G (2012) 'Dance Unit with Physical Literacy', *Procedia - Social and Behavioral Sciences*, 47:1135-1140, <https://doi.org/10.1016/j.sbspro.2012.06.791>

Anderson P, Anderson V and Lajoie G (1996) 'The tower of London test: Validation and standardization for pediatric populatons', *The Clinical Neuropsychologist*, 10(1):54-65, <https://doi.org/10.1080/13854049608406663>

Anico S, Wilson L, Eyre E and Smith E (2022) 'The effectiveness of school-based run/walk programmes to develop physical literacy and physical activity components in primary school children: A systematic review', *Journal of Sports Sciences*, 40(22):2552-2569, <https://doi.org/10.1080/02640414.2023.2174720>

Barbosa Filho VC, Pereira WMG, Farias BdO, Moreira TMM, Guerra PH, Queiroz ACM, Castro VHSd and Silva KS (2021) 'Scoping review on interventions for physical activity and physical literacy components in Brazilian school-aged children and adolescents', *International Journal of Environmental Research and Public Health*, 18(16), <https://doi.org/10.3390/ijerph18168349>

<https://www.mdpi.com/1660-4601/18/16/8349>

BOKS (2023) *BOKS Programming*, accessed 09/03/2023. <https://bokskids.org/program/>

Bopp T, Vadeboncoeur JD, Roetert EP and Stellefson M (2022) 'Physical Literacy Research in the United States: A Systematic Review of Academic Literature', *American Journal of Health Education*, 53(5):282-296, <https://doi.org/10.1080/19325037.2022.2100524>

Bremer E, Graham JD and Cairney J (2020) 'Outcomes and Feasibility of a 12-Week Physical Literacy Intervention for Children in an Afterschool Program', *International Journal of Environmental Research and Public Health*, 17(9), <https://doi.org/10.3390/ijerph17093129>

Caldwell HAT, Miller MB, Tweedie C, Zahavich JBL, Cockett E and Rehman L (2022) 'The impact of an after-school physical activity program on children's physical activity and well-being during the COVID-19 pandemic: a mixed-methods evaluation study', *International Journal of Environmental Research and Public Health*, 19(9), <https://doi.org/10.3390/ijerph19095640>

Canadian Sport for Life (2013) *Physical Literacy Assessment for Youth*, Canadian Sport Institute, Victoria, BC, Canada.

Carl J, Barratt J, Töpfer C, Cairney J and Pfeifer K (2022a) 'How are physical literacy interventions conceptualized?–a systematic review on intervention design and content', *Psychology of Sport and Exercise*, 58:102091.

Carl J, Barratt J, Wanner P, Töpfer C, Cairney J and Pfeifer K (2022b) 'The effectiveness of physical literacy interventions: A systematic review with meta-analysis', *Sports Medicine*, 52(12):2965-2999, <https://doi.org/10.1007/s40279-022-01738-4>

Carraro A, Young MC and Robazza C (2008) 'A contribution to the validation of the physical activity enjoyment scale in an Italian sample', *Social Behavior and Personality*, 36:911-918.

Chapelski MS, Erlandson MC, Stoddart AL, Froehlich Chow A, Baxter-Jones ADG and Humbert ML (2023) 'Parents, Teachers, and Community: A Team Approach to Developing Physical Competence in Children', *Children*, 10(8):1364, <https://doi.org/10.3390/children10081364>

Colella D and Bonasia M (2019) 'Teaching styles, physical literacy and perceived physical self-efficacy. Results of a learning unit in primary school', *Turk. J. Sports Med*, 54:1-7.

Coyne P, Vandenborn E, Santarossa S, Milne MM, Milne KJ and Woodruff SJ (2019) 'Physical literacy improves with the Run Jump Throw Wheel program among students in grades 4-6 in southwestern Ontario', *Appl Physiol Nutr Metab*, 44(6):645-649, <https://doi.org/10.1139/apnm-2018-0495>

Crocker PR, Bailey DA, Faulkner RA, Kowalski KC and McGrath R (1997) 'Measuring general levels of physical activity: preliminary evidence for the Physical Activity Questionnaire for Older Children', *Med Sci Sports Exerc*, 29(10):1344-1349, <https://doi.org/10.1097/00005768-199710000-00011>

CS4L (Canadian Sport for Life) (2014a) *PLAYfun Workbook*, Canadian Sport Institute–Pacific, accessed 9 March 2023. <http://physicalliteracy.ca/wp-content/uploads/2016/08/PLAYfun_workbook.pdf>

—— (2014b) *PLAYself Workbook*, Canadian Sport Institute–Pacific, accessed 9 March 2023. <http://physicalliteracy.ca/wp-content/uploads/2016/08/PLAYself_Workbook.pdf>

Deutsch J, Waldera R, Linker J and Schnabel E (2022) 'Impact of physical best warm-up activities on elementary students' physical activity levels and knowledge', *Physical Educator*, 79(4):424-440. <https://ezproxy.deakin.edu.au/login?url=https://search.ebscohost.com/login.aspx?direct=true&AuthType=ip,sso&db=s3h&AN=158178995&site=ehost-live&scope=site>

Dishman RK, Motl RW, Saunders R, Felton G, Ward DS, Dowda M and Pate RR (2005) 'Enjoyment Mediates Effects of a School-Based Physical-Activity Intervention', *Medicine & Science in Sports & Exercise*, 37(3):478-487, <https://doi.org/10.1249/01.Mss.0000155391.62733.A7>

Everley S (2021) 'Physical literacy and the development of girls’ leadership: an evaluation of the English Football Association’s Active Literacy through storytelling programme', *Education 3-13*, 50(5):668-683, <https://doi.org/10.1080/03004279.2021.1898433>

Farias C, Wallhead T and Mesquita I (2020) '“The Project Changed My Life”: Sport Education’s Transformative Potential on Student Physical Literacy', *Research Quarterly for Exercise and Sport*, 91(2):263-278, <https://doi.org/10.1080/02701367.2019.1661948>

Gavigan N, Belton S, Meegan S and Issartel J (2021) 'Moving Well-Being Well: a process evaluation of a physical literacy-based intervention in Irish primary schools', *Physical Education and Sport Pedagogy*:1-16, <https://doi.org/10.1080/17408989.2021.1967305>

Healthy Active Living and Obesity Research Group (HALO) (2017) *Canadian Assessment of Physical Literacy: Manual for Test Administration, 2nd Edn*, HALO, Ottawa, ON, Canada.

Heaton RK, Chelune C, Talley J, Kay GG and Curtiss G (1993) *Wisconsin Card Sorting Test Manual – Revised and Expanded*, Odessa, FL, USA.

Hulteen RM, Lubans DR, Rhodes RE, Faulkner G, Liu Y, Naylor PJ, Nathan N, Waldhauser KJ, Wierts CM and Beauchamp MR (2023) 'Evaluation of the peer leadership for physical literacy intervention: A cluster randomized controlled trial', *PLoS One*, 18(2 February), <https://doi.org/10.1371/journal.pone.0280261>

Invernizzi PL, Crotti M, Bosio A, Cavaggioni L, Alberti G and Scurati R (2019) 'Multi-Teaching Styles Approach and Active Reflection: Effectiveness in Improving Fitness Level, Motor Competence, Enjoyment, Amount of Physical Activity, and Effects on the Perception of Physical Education Lessons in Primary School Children', *Sustainability*, 11(2):405. <https://www.mdpi.com/2071-1050/11/2/405>

Jimenez-Garcia JA, Miller MB and DeMont RG (2023) 'Effects of multicomponent injury prevention programs on children and adolescents' fundamental movement skills: A systematic review with meta-analyses', *American Journal of Health Promotion*, 37(5):705-719, <https://doi.org/10.1177/08901171221146434>

Johnstone A, Hughes AR, Janssen X and Reilly JJ (2017) 'Pragmatic evaluation of the Go2Play Active Play intervention on physical activity and fundamental movement skills in children', *Prev Med Rep*, 7:58-63, <https://doi.org/10.1016/j.pmedr.2017.05.002>

Kozera T (2017) 'Physical literacy in children and youth', [Type], University of Manitoba Canada, accessed.

Kriellaars DJ, Cairney J, Bortoleto MAC, Kiez TKM, Dudley D and Aubertin P (2019) 'The Impact of Circus Arts Instruction in Physical Education on the Physical Literacy of Children in Grades 4 and 5', *Journal of Teaching in Physical Education*, 38(2):162-170, <https://doi.org/10.1123/jtpe.2018-0269>

Li MH, Rudd J, Chow JY, Sit CHP, Wong SHS and Sum RKW (2022) 'A Randomized Controlled Trial of a Blended Physical Literacy Intervention to Support Physical Activity and Health of Primary School Children', *Sports Medicine - Open*, 8(1):1-12, <https://doi.org/10.1186/s40798-022-00448-5>

Li MH, Sum RKW, Tremblay M, Sit CHP, Ha ASC and Wong SHS (2020) 'Cross-validation of the Canadian Assessment of Physical Literacy second edition (CAPL-2): The case of a Chinese population', *J Sports Sci*, 38(24):2850-2857, <https://doi.org/10.1080/02640414.2020.1803016>

Lightner J, Eighmy K, Valleroy E, Wray B and Grimes A (2023) 'The effectiveness of an after-school sport sampling intervention on urban middle school youth in the midwest: posttest-only study', *JMIR pediatrics and parenting*, 6, <https://doi.org/10.2196/42265>

Liu Y and Chen S (2021) 'Physical literacy in children and adolescents: Definitions, assessments, and interventions', *European Physical Education Review*, 27(1):96-112, <https://doi.org/10.1177/1356336X20925502>

—— (2022) 'Characterizing Middle School Students' Physical Literacy Development: A Self-Determination Theory-Based Pilot Intervention in Physical Education', *Frontiers in sports and active living*, 3:809447, <https://doi.org/10.3389/fspor.2021.809447>

Lloyd RJ (2016) 'Becoming Physically Literate for Life: Embracing the Functions, Forms, Feelings and Flows of Alternative and Mainstream Physical Activity', *Journal of Teaching in Physical Education*, 35(2):107-116, <https://doi.org/10.1123/jtpe.2015-0068>

Longmuir PE, Boyer C, Lloyd M, Yang Y, Boiarskaia E, Zhu W and Tremblay MS (2015) 'The Canadian Assessment of Physical Literacy: methods for children in grades 4 to 6 (8 to 12 years)', *BMC Public Health*, 15(1):767, <https://doi.org/10.1186/s12889-015-2106-6>

Longmuir PE, Gunnell KE, Barnes JD, Belanger K, Leduc G, Woodruff SJ and Tremblay MS (2018) 'Canadian Assessment of Physical Literacy Second Edition: a streamlined assessment of the capacity for physical activity among children 8 to 12 years of age', *BMC Public Health*, 18(2):1047, <https://doi.org/10.1186/s12889-018-5902-y>

Mandigo J, Lodewyk K and Tredway J (2019) 'Examining the Impact of a Teaching Games for Understanding Approach on the Development of Physical Literacy Using the Passport for Life Assessment Tool', *Journal of Teaching in Physical Education*, 38(2):136-145, <https://doi.org/10.1123/jtpe.2018-0028>

Marsh HW and Shavelson R (1985) 'Self-concept: Its multifaceted, hierarchical structure', *Educational Psychologist*, 20:107-123, <https://doi.org/10.1207/s15326985ep2003_1>

Mendoza-Muñoz M, Calle-Guisado V, Pastor-Cisneros R, Barrios-Fernandez S, Rojo-Ramos J, Vega-Muñoz A, Contreras-Barraza N and Carlos-Vivas J (2022) 'Effects of Active Breaks on Physical Literacy: A Cross-Sectional Pilot Study in a Region of Spain', *International Journal of Environmental Research and Public Health*, 19(13), <https://doi.org/10.3390/ijerph19137597>

Miyahara M (2020) 'Physical literacy as a framework of assessment and intervention for children and youth with developmental coordination disorder: A narrative critical review of conventional practice and proposal for future directions', *International Journal of Environmental Research and Public Health*, 17(12), <https://doi.org/10.3390/ijerph17124313>

Physical and Health Education Canada (2013) *Teachers guide: Passport for life*, accessed 9 March 2023. <http://passportforlife.ca/teacher/teachers-guide>

Pullen BJ, Oliver JL, Lloyd RS and Knight CJ (2020) 'The Effects of Strength and Conditioning in Physical Education on Athletic Motor Skill Competencies and Psychological Attributes of Secondary School Children: A Pilot Study', *Sports (Basel)*, 8(10), <https://doi.org/10.3390/sports8100138>

Pushkarenko K, Causgrove Dunn J and Wohlers B (2021) 'Physical literacy and inclusion: A scoping review of the physical literacy literature inclusive of individuals experiencing disability', *PROSPECTS*, 50(1):107-126, <https://doi.org/10.1007/s11125-020-09497-8>

Ryan RM (1982) 'Control and information in the intrapersonal sphere: An extension of cognitive evaluation theory', *Journal of Personality and Social Psychology*, 43:450-461, <https://doi.org/10.1037/0022-3514.43.3.450>

Santos S, Jimenez S, Sampaio J and Leite N (2017) 'Effects of the Skills4Genius sports-based training program in creative behavior', *PLoS One*, 12(2):e0172520, <https://doi.org/10.1371/journal.pone.0172520>

Saxena S and Shikako Thomas K (2020) 'Physical literacy programs for children with disabilities: a realist review', *Leisure/Loisir*, 44(2):199-224, <https://doi.org/10.1080/14927713.2020.1760119>

Stoddart AL, Humbert ML, Kerpan S, Cameron N and Kriellaars D (2021) 'PLitPE: an intervention for physical literacy enriched pedagogy in Canadian elementary school physical education classes', *Physical Education and Sport Pedagogy*:1-17, <https://doi.org/10.1080/17408989.2021.2014438>

Strobl H, Ptack K, Topfer C, Sygusch R and Tittlbach S (2020) 'Effects of a Participatory School-Based Intervention on Students' Health-Related Knowledge and Understanding', *Front Public Health*, 8:122, <https://doi.org/10.3389/fpubh.2020.00122>

Telford RM, Olive LS, Keegan RJ, Keegan S, Barnett LM and Telford RD (2021) 'Student outcomes of the physical education and physical literacy (PEPL) approach: a pragmatic cluster randomised controlled trial of a multicomponent intervention to improve physical literacy in primary schools', *Physical Education and Sport Pedagogy*, 26(1):97-110, <https://doi.org/10.1080/17408989.2020.1799967>

Ulrich DA (2004) *Test of Gross Motor Development 2nd Edition (TGMD-2)*, École des sciences de la réadaptation, Sciences de la santé, Université d’Ottawa, Ottawa, ON, Canada.

Wahyuni S, Lengkana AS and Sudirjo E (2023) 'Jump learning approach: a study on improving physical literacy of elementary school children', *JUARA: Jurnal Olahraga*, 8(1):315-329, <https://doi.org/10.33222/juara.v8i1.2601>

<https://jurnal.upmk.ac.id/index.php/juara/article/view/2601>

Wainwright N, Goodway J, Whitehead M, Williams A and Kirk D (2018) 'Laying the foundations for physical literacy in Wales: the contribution of the Foundation Phase to the development of physical literacy', *Physical Education and Sport Pedagogy*, 23(4):431-444, <https://doi.org/10.1080/17408989.2018.1455819>

Welk GJ, Corbin CB and Lewis L (1995) 'Physical Self-Perceptions of High School Athletes', *Pediatric Exercise Science*, 7:152-161.

Winsley RJ (2003) 'The Suitability of the Multistage Fitness Test to Assess Children's Aerobic Fitness', *European Journal of Physical Education*, 8(1):19-28, <https://doi.org/10.1080/1740898030080103>

Wright C, Buxcey J, Gibbons S, Cairney J, Barrette M and Naylor PJ (2020) 'A Pragmatic Feasibility Trial Examining the Effect of Job Embedded Professional Development on Teachers' Capacity to Provide Physical Literacy Enriched Physical Education in Elementary Schools', *Int J Environ Res Public Health*, 17(12), <https://doi.org/10.3390/ijerph17124386>

Zhang D, Shi L, Zhu X, Chen S and Liu Y (2023) 'Effects of intervention integrating physical literacy into active school recesses on physical fitness and academic achievement in Chinese children', *Journal of exercise science and fitness*, 21(4):376-384, <https://doi.org/10.1016/j.jesf.2023.09.004>
